# Mindfulness for people with chronic pain: Factors affecting engagement and suggestions for programme optimisation

**DOI:** 10.1111/hex.13745

**Published:** 2023-03-12

**Authors:** Fathima L. Marikar Bawa, Stewart W. Mercer, Jane W. Sutton, Christine M. Bond

**Affiliations:** ^1^ Institue of Applied Health Sciences, Centre of Academic Primary Care University of Aberdeen Aberdeen UK; ^2^ Stewart Mercer, Centre for Population Health Sciences, Old Medical School, College of Medicine and Veterinary Medicine, Usher Institute University of Edinburgh Edinburgh UK; ^3^ Department of Pharmacy and Psychology University of Bath Bath UK

**Keywords:** barriers and facilitators to engagement, chronic pain management, consensus techniques, mindfulness, psychological approaches, qualitative methods

## Abstract

**Introduction:**

Chronic pain is a common, multifactorial condition and pharmacological treatments have limited benefits. Mindfulness is a holistic approach that might be of value in the management of chronic pain. However, attrition rates from mindfulness‐based interventions are high and factors affecting engagement are unknown. The aim of this study was to inform the design of a mindfulness programme that would be accessible and acceptable for people with chronic pain.

**Methods:**

Interpretative phenomenological analysis of interview data from people with chronic pain who had taken part in an 8‐week mindfulness programme based on mindfulness‐based stress reduction revealed factors affecting engagement with and suggestions for tailoring the programme. Factors were grouped into physical, psychological and social domains. Further suggestions for tailoring the programme to address these factors were generated through a nominal group of healthcare professionals and a focus group with service users who had chronic pain.

**Findings:**

Physical factors included disability and discomfort with some practices; psychological factors included expectations of the mindfulness programme and understanding the relationship between mindfulness and pain; and social factors included loneliness and support from others. The proposed modifications to the mindfulness programme supported by healthcare professionals and/or service users to address these are described in this paper.

**Public Contribution:**

This study involved public contributions at a number of stages. The University of Aberdeen Division of Applied Health Sciences Service User Group (who were members of the public with chronic pain) was involved in the design of the study. Patients with chronic pain recruited from general medical practice who took part in the mindfulness programme were interviewed on their experience of the programme. Patients with chronic pain who attended the mindfulness programme, and healthcare professionals with expertise in chronic pain and/or mindfulness, attended meetings to design a tailored mindfulness programme for people with chronic pain.

## 1 INTRODUCTION

Chronic pain is common (prevalence estimates 20.4%^1^‐43.5%^2^), and can impact on, and be affected by, multiple aspects of patients' lives.^3^, ^4^, ^5^ The biopsychosocial model can be used to understand the experience of pain as an interaction of biological, psychological and social factors.^6^ Given the multifactorial nature of pain, pharmacological treatments have limited effect^6^, ^7^, ^8^ and a multidisciplinary approach using more than one treatment modality and including psychological or behavioural health approaches (such as cognitive behavioural therapy [CBT], acceptance and commitment therapy [ACT] and mindfulness‐based stress reduction [MBSR]) is recognised as being potentially beneficial.^9^, ^10^ CBT and ACT have been developed as part of treatments for chronic pain in recent years. However, these are often only available as part of pain management programmes offered in secondary care pain centres. The current National Institute for Health and Care Excellence (NICE) guidelines have suggested that there is some evidence of benefit from mindfulness, but not enough to make a recommendation, and have identified a need for further research.^10^ Mindfulness takes a different approach from CBT, with primary aims to enable a person to relate differently to their present experience (which may include physical and/or emotional discomfort) and cope better with their symptoms, rather than trying to control or change their symptoms.^11^, ^12^

Mindfulness, often taught as an 8‐week group programme^12^, ^13^ (e.g., MBSR), is increasingly used in self‐management of long‐term conditions.^14^ Tailoring mindfulness programmes to the needs of a particular population to increase accessibility, acceptability and potency, or orientating it around a cognitive model of a particular condition, has been encouraged.^12^ This has been done for people with recurrent depression in the United Kingdom and mindfulness‐based cognitive therapy (MBCT) is now a well‐recognised treatment for this population.^15^

Models for adapting mindfulness programmes for people with chronic pain include the Mindfulness for Health programme,^16^ and mindfulness‐based cognitive therapy for chronic pain (MBCT‐CP) combining the 8‐week mindfulness programme (based on MBSR) with CBT for pain.^17^ Pre–post data in general chronic pain populations show some indication of benefit.^18^, ^19^ Another approach combining ACT and mindfulness^20^ is currently at an early phase. The latter two approaches are based on theoretical models of pain.^21^ The majority of published randomised controlled trials (RCTs) of mindfulness for people with chronic pain continue to use the standard MBSR programme rather than a programme tailored to the pain population.^22^, ^23^, ^24^ To our knowledge, no studies have systematically looked at the barriers and facilitators to engagement with established mindfulness programmes based on MBSR to better understand the changes needed to improve acceptability for people with chronic pain.

Systematic reviews of mindfulness for people with chronic pain have shown high attrition rates from mindfulness programmes of up to 50% (median 20%).^25^, ^26^, ^27^ These are higher than those for the general public (nonclinical) where attrition rates are up to 35%, (median 11.5%),^28^ but reasons for this are unknown. Studies have reported practical and logistical reasons for dropping out^29^, ^30^, ^31^, ^32^, ^33^, ^34^, ^35^, ^36^, ^37^, ^38^, ^39^, ^40^ and other external factors such as family obligations^29^, ^30^, ^32^, ^35^, ^39^, ^40^ but only a few have looked at psychological factors such as belief in the treatment,^41^ or motivation to continue practicing,^33^, ^42^ with no studies exploring factors affecting engagement as an objective.^43^

Poor attendance at psychological therapies has been described as an indicator of nonengagement which can affect intervention effectiveness.^44^ Engagement with mindfulness programmes has been described as developing an approach that would be sustained beyond completion of the programme with formal and/or informal practices of mindfulness.^45^ Holdsworth et al. defined engagement with psychological therapies as consisting of attendance, homework completion, involvement (made up of motivation, belief, commitment and intent) and therapeutic relationship.^44^ Banerjee et al. applied this specifically to mindfulness‐based interventions, suggesting that engagement involves physical engagement (session attendance and between‐session mindfulness practice), psychological engagement (consisting of motivation to participate in the programme, the belief that practicing mindfulness will be beneficial, commitment to bringing mindfulness into their daily life and intention to maintain a mindfulness practice) and the therapeutic relationship between the participant and the programme facilitator and group.^46^ This recognition of the physical, psychological and social aspects of engagement are taken forward in this paper.

We conducted a multiphase feasibility study on delivering a mindfulness programme to people with chronic pain.^43^, ^47^ Members of the University of Aberdeen Division of Applied Health Sciences service user group for people with lived experience of chronic pain were consulted at two meetings in the early stages of the research to contribute to the design of the study. Participants were recruited from general medical practices in Fort William, a small town in the Highland Health Board area of Scotland. Eligibility criteria were: >18 years old; having nonmalignant chronic pain (≥3 months). Patients were identified from a general practitioner (GP) prescribing records.

The mindfulness programme (Supporting Information: Appendix 1), based on MBSR^48^ took place in October/November 2013. This included 8‐weekly sessions covering the standard core components (sitting meditation, body scan meditation, mindful movement and daily home practice).^12^, ^13^ The Mindfulness Scotland workbook^47^ (also available from Mindfulness Scotland), based on the standard MBSR curriculum,^12^, ^13^, ^48^ provided weekly handouts and home practice resources.

Preprogramme interviews were conducted face‐to‐face by the programme facilitator (S. R.) during which the programme was described, questions addressed and written consent taken.^49^ Interviews were conducted face‐to‐face by the lead researcher (F. L. M. B.) postprogramme, and by telephone 8 months after programme end by F. L. M. B. All interviews were audio‐recorded and transcribed verbatim.

Based on our earlier systematic review,^27^ participants were categorised as nonattenders (did not attend a single session), noncompleters (attended between 1 and 3 sessions) and completers (attended a minimum of four of the eight sessions). Data were stored and managed using NVivo v10 software. Interview recordings were listened to while reading the transcript several times, with subsequent coding and theming of the interview data by F. L. M. B. using interpretative phenomenological analysis (IPA).^50^ One transcript was double‐coded by an experienced IPA researcher (J. W. S.), differences resolved by discussion, and themes iteratively reviewed.

The findings from IPA analysis for the 34 participants who took part in the mindfulness programme are reported in a separate paper.^47^ Findings comprised factors affecting experience (influence of earlier life events; the process of taking part in, and of relating to, the programme); and effects of the programme (impact on emotions, mental health; adverse events and a process of change). Factors affecting the experience included expectations of pain relief and other preconceptions. Understanding of the objective of the programme seemed to occur around week 4. Effects of the programme included the process of change, which occurred only once there was an understanding of the relationship between mindfulness and pain (this understanding developed as they began to incorporate mindfulness into their lives). The process of change involved learning to ‘listen to the body’, gaining a sense of community, learning to accept pain and approaching life with more self‐care, awareness, appreciation and empowerment. Greater self‐awareness through the process of change led to taking greater responsibility for their feelings and health.

There is a need to identify the experiences that affect engagement with the mindfulness programme, link these to particular stages of the programme and use them to inform future modifications. It is important to ensure people with lived experience of chronic pain as well as people with expertise in treating chronic pain and/or teaching mindfulness are involved in this process. This may help reinforce existing models tailoring the programme for people with chronic pain and suggest other areas that may be important to consider.

### 1.1 Study aims

The aim of this paper is to explore factors affecting engagement with and propose modifications to the mindfulness programme with the intention of improving accessibility and acceptability for people with chronic pain.

## 2 METHODS

We drew on the datasets from the study described in the introduction. These comprised the following: preprogramme interviews (D1), postprogramme interviews (D2) (see Supporting Information: Appendix 2, for the interview topic guide) and longitudinal follow‐up interviews (D3). The interviews explored participant expectations and experiences; and at postprogramme and longitudinal follow‐up they included asking how the programme could be changed to be more acceptable/practical for them.

### 2.1 Identifying factors affecting engagement

Themes that represented barriers and/or facilitators to engagement at the different stages of the programme together with suggestions for modifications identified in the previous IPA analysis, were extracted by two co‐authors (F. L. M. B. and C. M. B.). They grouped the barriers and facilitators into physical, psychological and social factors using the following definitions. Physical factors were defined as those experienced physically (such as disability) or practically (such as transport). Psychological factors were defined as cognitive experiences (motivation, mental health and emotions) and social factors as those that depend on others (such as family support). Where a factor was influenced by more than one of these domains, this was reflected in the label: socio‐physical (a factor, i.e., both social and physical such as exclusion due to disability), psycho‐physical (a psychological and physical factor such as listening to the body), psycho‐social (a psychological and social factor such as the impact of the group on wellbeing) or psycho‐socio‐physical (a factor that incorporates all three domains such as spirituality) are used. The factors affecting engagement and suggestions for modification were matched to the stage of the programme that they referred to (enrolment, attendance, doing home practice and continued mindfulness practice after programme completion), and were then taken forward to the subsequent phases.

### 2.2 Generation of list of programme modifications

There were two phases to the modification generating process. The first phase involved a nominal group meeting^51^ with healthcare professionals with expertise working in the field of mindfulness and/or chronic pain to get consensus on the modifications to take forward to the second phase; a focus group (D4) with service users (people with lived experience of chronic pain who had attended the mindfulness programme) to identify further factors affecting engagement and suggestions for modifications. A final list of modifications was then compiled, combining healthcare professional and service user suggestions using an iterative process (Figure 2). Details of these two phases are described below.

#### 2.2.1 Phase 1

**Figure 1:**
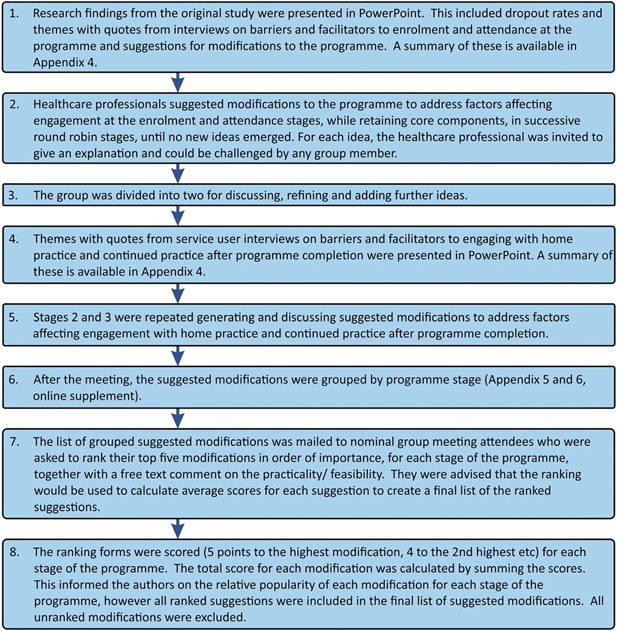
Phase 1: Nominal group.

Potential participants for the nominal group meeting were identified through purposive sampling to include representation from a range of different healthcare professions and balance of genders. They were selected from the personal networks of one of the co‐authors (S. W. M.) who is a medical practitioner, academic and member of Mindfulness Scotland, and 17 were sent an invitation letter, information sheet and consent form by email. There was a recruitment target of seven.^51^ Before the meeting, those who had consented to attend were sent a summary of the research findings, the Mindfulness Scotland Workbook and mindful movement handouts that were used for the programme. The meeting was held in Glasgow in November 2017, facilitated by two of the authors (F. L. M. B. and C. M. B.) and audio‐recorded. Details of the meeting are shown in Figure 1 below.

#### 2.2.2 Phase 2

**Figure 2:**
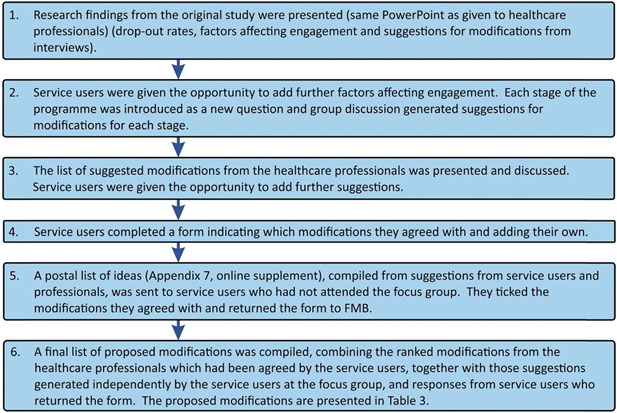
Phase 2: Focus group and compiling the final list of ideas.

All original study participants who had attended one or more mindfulness sessions (*n* = 24) were telephoned by the lead author (F. L. M. B.) to invite them to the focus group meeting to discuss modifications to the mindfulness programme; and to assess their availability and preferences for timings of the meeting. Depending on the number available, one or two focus group meetings would be arranged (so that no more than 12 participants attended each meeting). The group size was considered to be large enough to enable discussion but not so large that it would prevent some members from sharing insights in the available time.^52^ Based on this principle, the target number to attend each group was eight, allowing for some cancellations.^52^ The original participant information sheet and consent form used when recruiting participants to the overall study had included information about the focus group meeting and stated that it would be audio‐recorded. Those who were interested in attending the meeting and available on the suggested dates were then mailed a letter giving information on the timing, date and location of the meeting and describing what would happen. The meeting was held at Fort William Health Centre in December 2017, facilitated by F. L. M. B. and audio‐recorded. Details of the meeting are shown in Figure 2 below.

## 3 FINDINGS

### 3.1 Recruitment and retention

#### 3.1.1 Interview participants

Thirty‐four participants consented to take part in the mindfulness programme of whom 24 attended one or more sessions (10 noncompleters and 14 completers). Of the 34 participants who consented to take part, most were female (27/34), aged between 50 and 73 years (24/34) (most common age group 58–65 years [10/34]), Christian [22/34] and all were Caucasian. The most common pain diagnoses were osteoarthritis (12/34), mechanical back/neck pain (4/34), rheumatoid arthritis (4/34) and fibromyalgia (3/34). The average pain score on a scale of 1–10 (1 = least, 10 = most) over the 2 weeks before the programme was 6.5 (completers 5.9 and noncompleters 7.6).

#### 3.1.2 Nominal group meeting

Seven healthcare professionals (7/17) attended the nominal group meeting (Table 1). All seven completed the ranking form.

**Table 1:** Demographics of healthcare professional attendees.

Background	Gender	Healthcare Professional	Mindfulness teacher	Mindfulness expert
Clinical psychologist	Male	✓	✓	✓
Clinical psychologist	Female	✓	✓	✓
Consultant clinical psychologist and national pain management programme clinical lead	Female	✓	✓	
Consultant in pain medicine	Male	✓	✓	
General practitioner	Male	✓		✓
Consultant psychiatrist	Male	✓	✓	✓
Academic researcher and cancer nurse	Female	✓		✓

#### 3.1.3 Focus group and postal list of ideas

**Figure 3:**
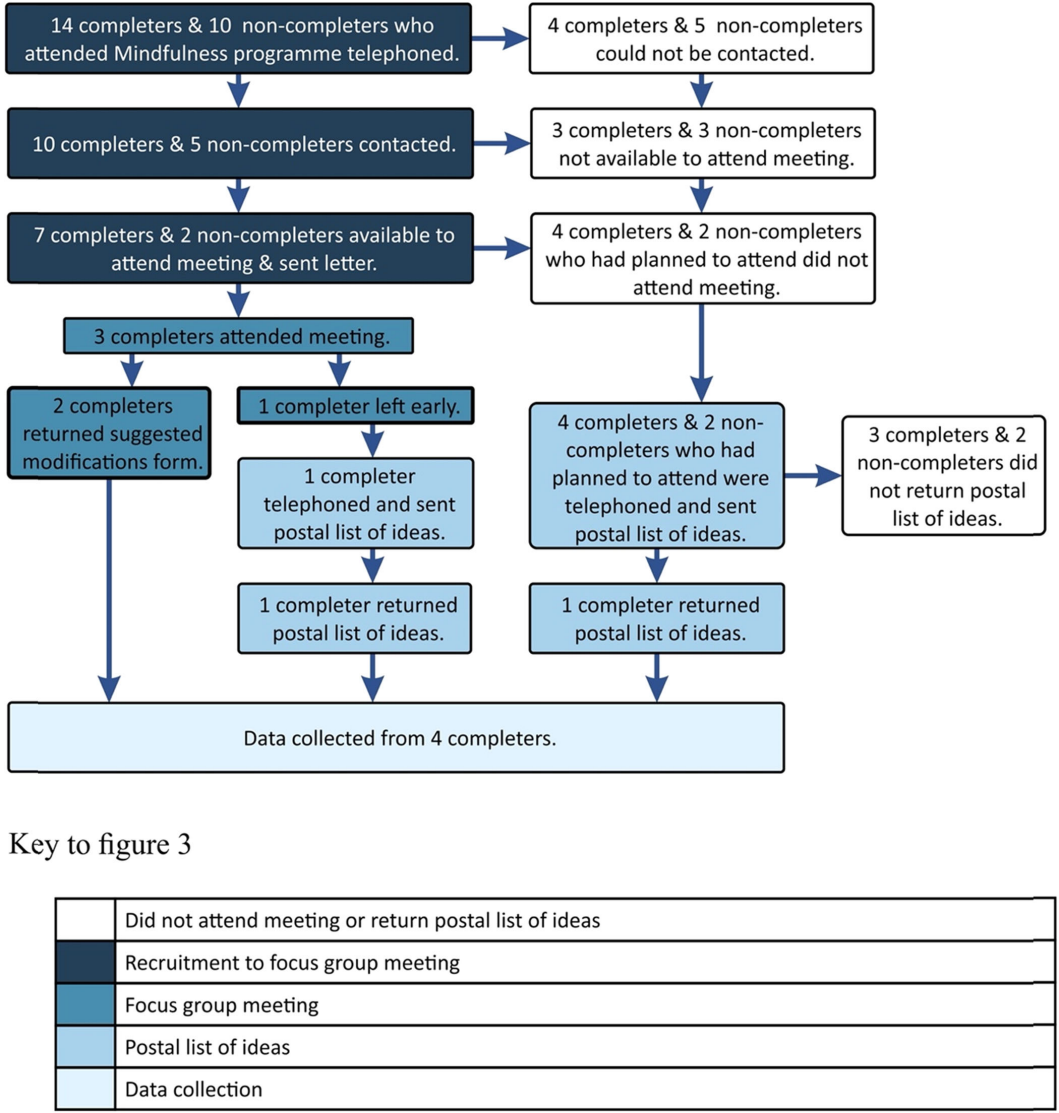
Flowchart of participants who attended focus group and/or returned postal list of ideas.

Four service users attended the focus group meeting and/or returned the postal list of ideas (Figure 3).

#### 3.1.4 Factors affecting engagement with the mindfulness programme

**Figure 4:**
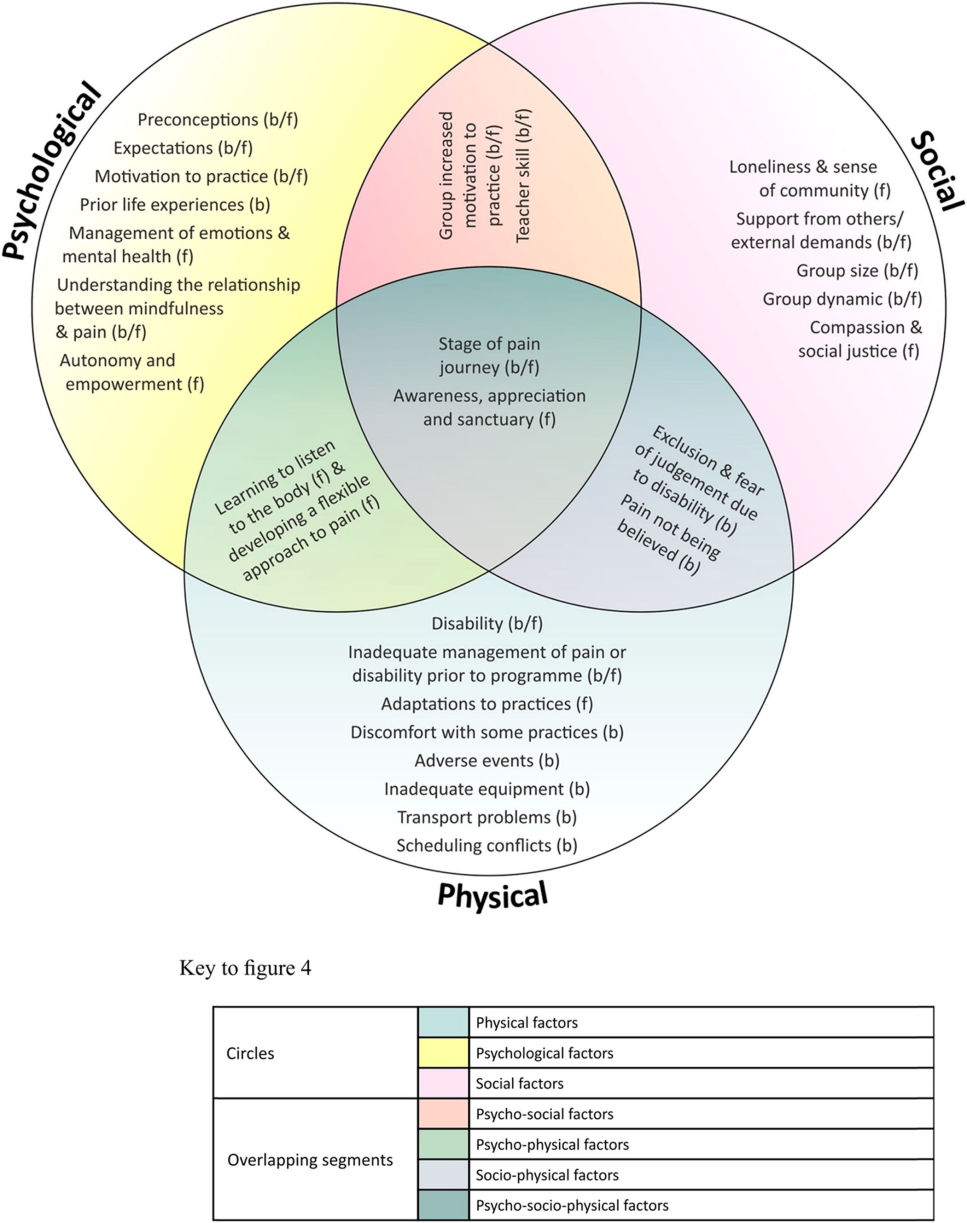
Factors affecting engagement are grouped into psychological, social, and/or physical domains. b, barrier; f, facilitator.

Factors affecting engagement grouped into psychological, social, and/or physical domains, identified from preprogramme interviews (D1), postprogramme interviews (D2), longitudinal follow‐up interviews (D3) and the focus group (D4), are shown in Figure 4. Some were barriers to engagement, some facilitators and some could be either.

In Table 2, factors affecting engagement with the programme are listed, with quotes, grouped into physical, psychological and/or social domains, as illustrated in Figure 4. Supporting Information: Appendix 3 shows how the factors affecting engagement relate to each stage of the programme (enrolment, attendance, home practice and continued practice). One of the factors, ‘stage of pain journey’, emerged during the focus group meeting (D4). Participants described the pain journey as the course of a chronic pain condition, with various stages of learning, seeking treatment and adapting. They expressed that mindfulness may be appropriate at a particular stage of the pain journey.

**Table 2:** Factors affecting engagement.

Type of factor	Factor	Quote	Data source	Participant ID, completer status
Physical	Disability	‘My pain kicks in when I'm standing or walking, um, so when they were asking people to do, like walking about the room, um, I said, “look, I'd prefer not to” [….] I sat down, and she says “well”, er, “try doing it and moving your feet”, I says “look I can't do that either, that's restrictive for me as well”, so I did feel quite limited as to what I could take part in, really to be honest’.	D2	T194, noncompleter
	Inadequate management of pain or disability before programme	‘Half of those people can't feel anything, they are so numb on the drugs they're on and they are in so much pain, and their posture is so distorted. Walking around, being mindful of your body is um, that's 3 years down the line, that's, that's not for a class like that, they need physical treatment before they can start getting into that level of mindfulness’.	D2	G59, noncompleter
	Adaptations to practices	‘When they indicated they were going to have some meditation, I said well “How long is this going to take? I can be here for five minutes, or 10 minutes, but any longer than that”, and they got to say, “Right, XXXX [participant name], you'll need a mat for this one” [….] I was quite happy really, I had my mat to lie on the floor, that was really all I needed’.	D2	T44, completer
	Discomfort with some practices	‘The body scans, which I found very difficult on my own [….] I've got used to, if you like, putting it to one side, and dealing with it, it was bringing it to the front again, and in a way, making it worse, I know this sounds silly, making it worse, um, so I've had a lot more trouble with it’.	D2	G87, completer
	Adverse events	‘Where I live I look onto XXXX [mountain name], and you know, you're meant to do mindful thinking, so I thought, “Okay”. I was standing just thinking, “What a wonderful view”, and all the things around it, forgetting that I've got a little dog on the end of the lead, and I went smack, straight over the dog, because I wasn't paying attention to where I was walking’.	D2	G165, completer
	Inadequate equipment	‘Your chairs, were horrendous [….] so it got to the stage, it was causing more pain in going to the course, because the chairs were so uncomfortable, very uncomfortable, and I, I think the girls would have said that themselves, I don't think any of us didn't say that that was the wrong type of chair’.	D2	T165, completer
	Transport problems	‘I'm not sure. Two hours, mmmh, that time of night as well, it means me driving there and back as well, of course, because there is no bus service at that time. I don't, the time doesn't suit me, that time, it will be dark almost, but we will see’.	D1	G112, nonattender
	Scheduling conflicts	‘The time that you're picking is tea time for most people’.	D1	T165, completer
Psychological	Preconceptions	‘I obviously heard about meditation, but I always thought that, you know, “What good can it do me?” [….] You see things on the TV, but you always see people sitting, you know, on the floor [….] and humming away, and I thought, “Mmmh, I don't know if that would ever be for me”, but er, but this wasn't so much like you see it on the TV, kind of thing, it was more accessible I think’.	D2	G23, completer
	Expectations	‘I was hoping that it was something that was going to relieve my pain, other than using pain killers [….] when it said the class was for people with chronic pain, I thought that. [….] I did, um, realise after I'd been the third time, I think, I thought, “I don't think this is about pain relief after all!” [laughs]’.	D2	T215, noncompleter,
	Motivation to practice	‘The first 4 weeks were like a wee voyage of discovery [….] it's almost as though there was the class happening and that's where everything happened, you came home, you did the exercises, but that's all they really were, and then about week 4, you actually started incorporating, well I started incorporating it into, not just my room, and, and the session, but in other places and at other times’.	D2	G47, completer
	Prior life experiences	‘When I was a child, it would be more stiffness and er<just not able to do the same as all the other kids>, I, I, I always wanted to do everything just the same [….] but everything was just stiffer, you know, because all the tendons were just so tight. [….]. My pain kicks in when I'm standing or walking, um, so when (at the mindfulness programme) they were asking people to do, like walking about the room, um, I said, “look, I'd prefer not to”, and I just sat down [….] And then there were other exercises they were getting people to imagine there was a, a barrel and they were pushing it, and there were a lot of people were standing to do it, so I sat down, and she says “well”, er, “try doing it and moving your feet”, I says “look I can't do that either, that's restrictive for me as well”, so I did feel quite limited as to what I could take part in, really to be honest’.	D2	T194, noncompleter
	Management of emotions and mental health	‘I've been able, a few times, sort of in stressful situations at work [….] the breathing spaces [….] that's been quite invaluable, um, being able to sort of realise that I'm about to burst into tears or cry or do something silly, um, and so take a few minutes, and [pause] I should be able to get back in control, and manage things’.	D2	G21, completer
	Understanding the relationship between mindfulness and pain	‘I just found it really strange, and I wasn't quite sure what the object of it was [….] I just didn't understand some of the exercises we were doing and why we were doing them [….] I don't want to focus on my pain, you know, if, because I think if I did, then ‘I would be a bit depressed, I think, you know, so I couldn't quite follow why they were wanting us to focus on it’.	D2	T194, noncompleter
	Autonomy and empowerment	‘When you do become aware of what's happening [….] you can see where you were going wrong [….] and you can also see how mean you've become and so to move on, you have to recognise that that was part of you […] being honest to yourself, is the biggest thing about mindfulness. [….] I've been talking to the physiotherapist about er, doing acupuncture, which I think would be of great benefit to me [….] I'm going out walking much more, which is very good for me, [….] from an emotional point of view, I'm setting up the painting and having my own studio [….] the art for me, that's my, that's my spiritual thing I think, um, I'm definitely continuing with mindfulness’.	D2	T264, completer
Social	Loneliness and sense of community	‘I had got to feeling quite alone about it, and quite beyond anybody helping me [….] and then somebody else made a comment as well, and I thought, “Perhaps there are other people here who probably with their pain and everything else, do feel DESPERATELY alone, because their partners or their friends, don't understand what it is like” [….] unless you've had back pain, you don't understand, you haven't got a clue [….] THAT was quite poignant to me, you know, and the woman said to me, “Yeah, you're not alone”, so, yeah’.	D2	G87, completer
	Support from others/external demands	‘I think they smile and, and, I think they're quite pleased, I think they're quite pleased for me, you know, there's a bit teasing goes on, but you know, “What's mum's, what's mum's target this week” [….] if I'd been on my own, I don't think I would have gone [….] I think having people around you to say, “Oh this is the night that you're going, and what time are you going at”, you know, so that wee support at home, and it was support really [….] that did help’.	D2	G47, completer
	Group size	‘Maybe if the group wasn't so big?, I mean I know it had to be, it had to be big, um, but I mean, if it was maybe like two separate groups?, I, maybe I'd cope better with that, but because there was so many people [….] something like 12, yeah, whereas, the group was so big, you couldn't really get to know anybody [….] whereas in a smaller group, I think it's easier to get to know people’.	D2	G74, noncompleter
	Group dynamic	‘There's a lot of trust in the group as well, so that, that was, a little, a sort of little base [….] if somebody had had a good week, or had a good experience, then you all felt, you know it's a sort of shared happiness, or shared enthusiasm, and it was, you know, we all shared in it [….] it was quite open, you really felt you could express yourself, and, and people understood [….] and I found it really, I found it really, really useful, not just from, you know, just benefitting from other people's experiences as well’.	D3	G47, completer
	Compassion and social justice	‘I think it makes you mindful, it doesn't make you think of a religion, or a faith, or anything like that, it makes you mindful that you have to look after yourself, you have to look after the love that you have for other people and for yourself, it makes you mindful of, yes, of the world and that, you know, recycling's where possible a pretty good thing, and you know, not going to war might be a good thing too, you know. Making good decisions, where you can, but also recognising you're just a wee speck in the planet, that's been going for millions of years’.	D2	T264, completer
Psycho‐physical	Learning to listen to the body and developing a flexible approach to pain	‘I think I just get more and more tense, just more and more uptight, uptight, but when you start feeling yourself, you know, knotting, you just take a wee bit of time out, so I've seen me doing that in bed [….] just lie on my back and just basically I start with the toes, and work my way up, and just, just concentrate on relaxing, basically’.	D2	T181, completer
Psycho‐social	Group increased motivation to practice	‘I can do what we were doing in the group, but on my own, but I'm much more easily distracted, because I'm not out of life, in a purpose‐built situation [….] I just can't do it on my OWN! I've TRIED, God knows I've tried, it just DOES NOT WORK!’.	D2	G87, completer
	Teacher skill	‘It was nice to see that er, she and XXXX [facilitator 2] were always very calm, and I thought, “Well that's quite reassuring” [….] they could disagree with something that somebody had said, without being at all confrontational [….] “That's how you see it, and not everybody sees it the same” [….] they take the time to just be calm about it, and think about it, this is definitely a better way of working, you know?’	D2	T214, completer
Socio‐physical	Exclusion and fear of judgement due to disability	I just felt like, you know, “Oh if I sit down, people will be thinking I'm at it”, you know, so you try and sort of PUSH yourself [….] people were sort of looking at you, and when people look at me, I tend to want to go in a corner and hide’.	D2	G74, completer
	Pain not being believed	‘I've been ‘treated as a psychiatric case [….] I came here to ask for help from GP's (for spinal injuries) [….] they basically told me there was nothing wrong with me and offered me Prozac [….] I've lost faith in GPs. There's a lack of awareness of physical pain [….] too much emphasis on the mental health side of pain’.	D1	G59, noncompleter
Psycho‐socio‐physical	Stage of pain journey	‘I had reached a point where life was diminished due to incapacity and pain [….] I couldn't go on [….] If I had been invited at an earlier point I might have thought, “I can't cope with that (the mindfulness course), I'm too tired” [….] I might not have seen the value of it [….] so it was the right time’.	D4	T264, completer
	Awareness, appreciation and sanctuary	‘I think the cemetery is so peaceful and that, you just, the, I love the autumn colours anyway, just, even when you look, you've got all the trees around you, up in XXXX [area], the shapes of the trees you're more aware of it, and then you hear the birds. [….] (Before the programme) you just sort of walk up, go visit, put flowers down, and walk away, and that, but you just take your time now, go way up and just, you're more absorbing, in, you know, your environment and [….] I think I'm paying more attention now, because I just love it, because even I'll say to dad, “Just sit and listen to the birds, and how nice and peaceful it is”, just trying to get him better aware’.	D2	T181, completer

*Note*: T#, participant ID number; D#, data source (D1–D4); capitalised text, verbal emphasis is given by the interviewee; [….], text omitted for brevity and XXXX, text omitted for confidentiality

Abbreviation: GP, general practitioner.

#### 3.1.5 Suggested modifications to the mindfulness programme

The final list was made up of 20 suggested modifications to the programme, which are displayed in Table 3. Justification is given for each modification and the factor(s) affecting engagement being addressed by the modification is shown. Eighteen of the proposed modifications were supported by both groups. One was suggested by service users at the focus group meeting and not discussed with healthcare professionals due to the time sequence of the meetings. One was suggested by healthcare professionals and not discussed with service users due to time constraints and because it would only affect the behaviour of the healthcare professional rather than the service users themselves.

**Table 3:** Proposed modifications.

Modification (suggested by healthcare professionals (P)/service users (S))		Supported by	Stage of programme	Factor addressed	Justified by healthcare professionals (P)/service users (S)/interview finding (I)
1	Mindfulness to be recommended and supported by GP or other healthcare professional providing ongoing support (P)	Healthcare professionals. Not discussed with service users	Enrolment	Support from others	Importance of personal approach to enroling and ongoing support. (P)
2	Preprogramme letter explaining the objective of the programme (changing the relationship to pain and improving quality of life) in accessible lay language with testimonials, infographics and links to patient stories (P)	Both groups	Enrolment	Expectations	Communicating programme objectives would help manage expectations. (I)
3	Mindfulness taster session before screening interview (to give an orientation on mindfulness including information on its use in health care and experience of mindfulness practices) (P)	Both groups	Enrolment	Preconceptions Pain not being believed Expectations Understanding the relationship between mindfulness and pain	Several participants did not understand the objective of programme. (I) Could involve graduates of programme with chronic pain. (P) To be better informed before enroling. (S)
4	Screening interview: enrol/redirect according to where the patient is on their pain journey and what they want to achieve (P, S)	Both groups	Enrolment	Stage of pain journey Expectations Inadequate management of pain or disability before programme	Physical treatment for pain may be needed before mind‐body approaches. (S) Chronic pain is experienced as a ‘journey’ involving learning, seeking treatment, and adapting to living with a condition. Mindfulness is appropriate at some, not all, stages of the journey. (S) Redirection to pain clinic/physiotherapy if more appropriate. (P/S)
5	Screening interview, conducted by an experienced mindfulness teacher who is teaching the programme, to explain the objective of programme, time required and that it may take a few weeks to benefit (P)	Both groups	Enrolment	Expectations Understanding the relationship between mindfulness and pain	Important to managing expectations early. (P/S) Several participants did not understand the objective of programme. (I) Some dropped out due to unmet expectations around pain relief. (I)
6	Equipment: provide appropriate equipment (chairs, mats, blankets) (P)	Both groups	Attendance	Disability Discomfort with some practices Inadequate equipment	Some may attend straight from work, bringing equipment may be a barrier. (P)
7	Teachers with adequate training, experience, skill and personal practice of mindfulness (P, S)	Both groups	Attendance	Teacher skill Understanding the relationship between mindfulness and pain Support from others	Teachers skill at leading the inquiry and relating it to pain important. (P/S) Teachers embody mindfulness through having a personal practice is important. (P/S)
8	Limit group size to a maximum of 12–15 people (P)	Both groups	Attendance	Loneliness and sense of community Group size (smaller groups enabled a better sense of community)	Several found the group size (24) too large, and preferred the smaller group as the programme progressed. (I)
9	Tailor content of the programme to pain: give pain relevant information and practices (P)	Both groups	Attendance	Understanding the relationship between mindfulness and pain Pain not being believed	Mindfulness‐based cognitive therapy^53^ has exercises on working with thoughts and feelings. The equivalent for pain should be included in programme. (P)
10	Build up the duration of meditation practices gradually but keep the longer (30 min) practices in (P)	Both groups	Attendance	Disability Discomfort with some practices	Benefit in staying with what is difficult. (P/S) Deeper meditation with longer practices. (S)
11	Demonstrate two or three levels for each exercise for participants to choose which is most appropriate (P, S)	Both groups	Attendance	Disability Adaptations to practices Exclusion and fear of judgement due to disability Learning to listen to the body and developing a flexible approach to pain Autonomy and empowerment	Participants felt self‐conscious opting out of exercises due to disability. (I) Giving options reinforces self‐care and self‐responsibility. (P) Choosing from two or three options felt empowering. (S)
12	Acknowledge people's pain and encourage doing what is needed to manage pain during practices (e.g., stand up/move/lie down) (P)	Both groups	Attendance	Exclusion and fear of judgement due to disability Pain not being believed Autonomy and empowerment	Some felt they were not believed for being in pain. (I) Some practices made their pain worse but afraid of being judged if opted out. (I) Would encourage responding flexibly to pain. (P)
13	Support between sessions: opt in to receive reminder texts to be mindful (P)	Both groups	Home practice	Support from others	
14	Support line for people to call/email between sessions if needed (P)	Both groups	Home practice	Support from others	Support from teachers with home practice difficulties was helpful. (I) Opportunity to discuss adverse events or support adaptations to practices. (P)
15	Home practice recordings specifically for pain and having two or three options for different levels of ability and of different lengths of time (P)	Both groups	Home practice	Disability Learning to listen to the body and developing a flexible approach to pain Autonomy and empowerment	Pain‐relevant programme important. (S) Having three different levels would enable choice. (S)
16	Inquiry session at weekly group meeting specifically asking about home practice (P)	Both groups	Home practice	Support from others Discomfort with some practices Adverse events Adaptations to practices	Inquiry session carried out in a nonjudgemental way: generate ideas for overcoming obstacles to home practice. (P)
17	Have all‐day session during programme open to people who have completed the programme, serving as refresher session for completers (P)	Both groups	Continued practice	Pain not being believed Support from others	Validate relevance of programme to people with pain. (P) Improve motivation to continue to practice. (P/S)
18	Build follow‐up group into programme (P)	Both groups	Continued practice	The group increased motivation to practice Loneliness and support from others	Difficult to practice alone/without reinforcement of a regular class. (I) Enable ongoing practice together after completion of programme. (I)
19	Use local community and online resources to support ongoing practice (P)	Both groups	Continued practice	Loneliness and support from others Group increased motivation to practice	Feasible if using existing websites/resources. (P)
20	Encourage participants to develop their own resources for ongoing practice (S)	Service users. Not discussed with healthcare professionals	Continued practice	Autonomy and empowerment Adaptations to practices	Could be tailored to the individual, for example, music listened to mindfully, time in nature. (S)

*Note*: (P), suggested by healthcare professionals at the nominal group meeting; (S), suggested by service users at the focus group meeting and (I), findings from interviews with service users.

Abbreviation: GP, general practitioner.

**Figure 5:**
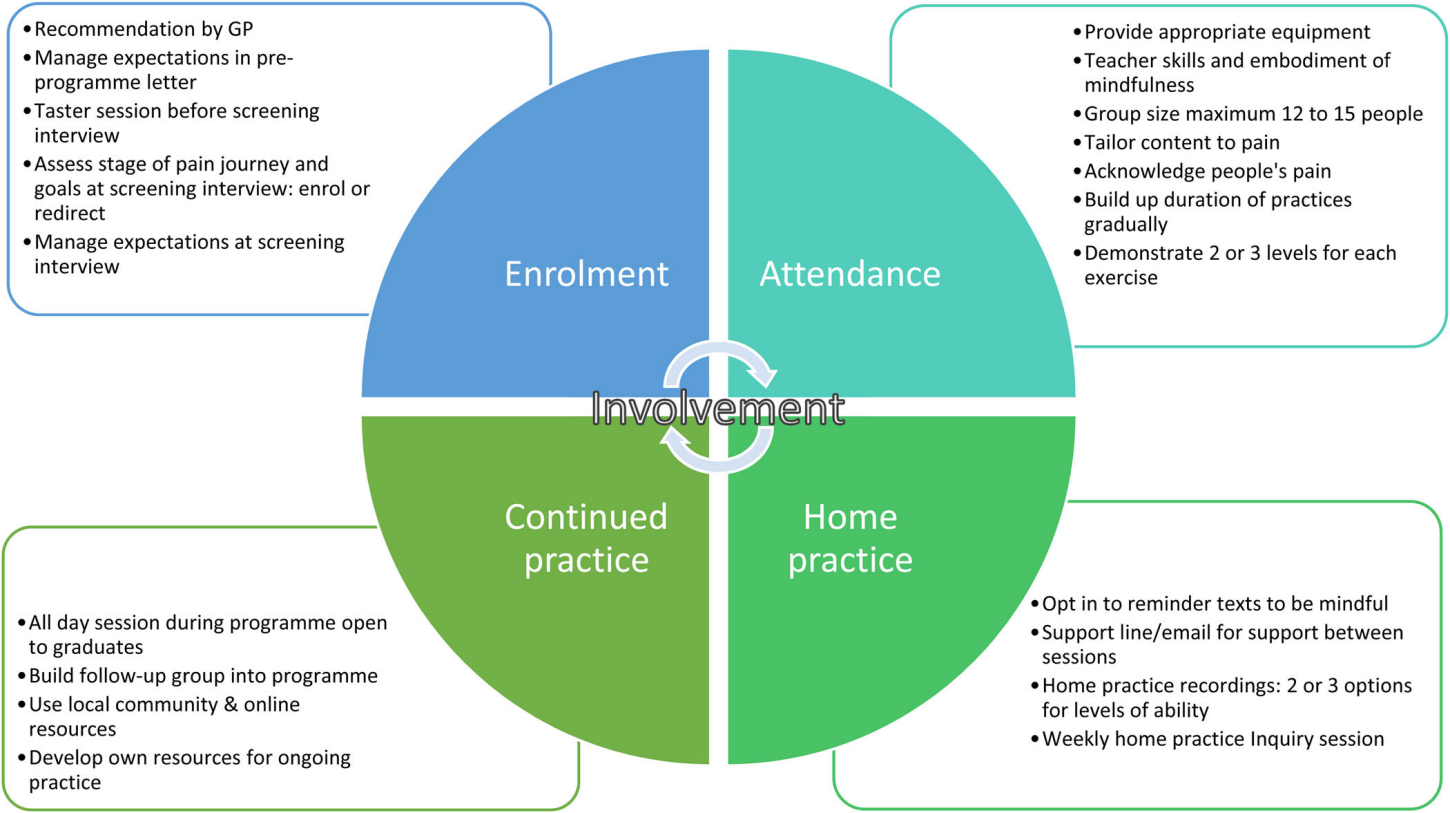
Modifications to programme to promote engagement. GP, general practitioner.

Figure 5 shows which stage of the programme each of the 20 suggested modifications apply. These are thought to increase involvement (psychological engagement) and taken together to increase overall engagement with the programme.

**Figure 6:**
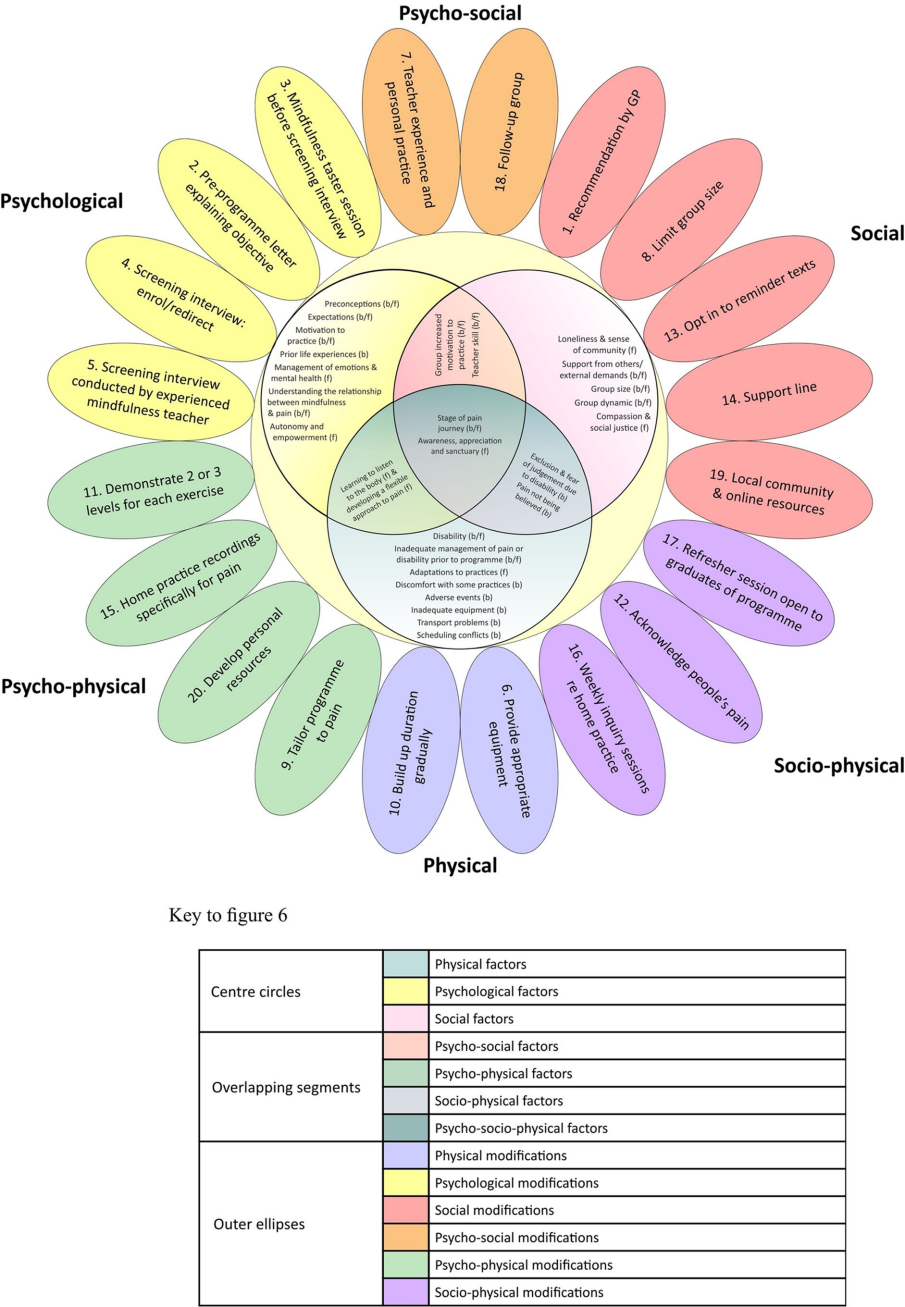
Modifications to address factors affecting engagement per domain.

In Figure 6, suggested modifications are grouped into physical, psychological and/or social domains with corresponding factors affecting engagement.

## 4 DISCUSSION

### 4.1 Summary of main findings

Factors affecting engagement with a mindfulness programme for people with chronic pain were identified and suggestions for modifying the programme were generated. Physical factors (e.g., inadequate management of pain or disability before embarking on the programme), psychological factors (e.g., understanding the relationship between mindfulness and pain) and social factors (e.g., developing a sense of community) were identified. Factors were also identified where physical, psychological and social domains overlapped, including psycho‐physical factors (e.g., developing a flexible approach to pain), psycho‐social factors (e.g., teacher skill), socio‐physical factors (e.g., the experience of pain not being believed) and psycho‐socio‐physical factors (e.g., stage of pain journey). The ‘pain journey’ was recognised as an experience of a chronic pain condition over time, involving various stages of learning, seeking treatment and adapting to living with a condition, with mindfulness potentially appropriate at some, but not all, stages of the journey.

Twenty suggestions for modifying the programme to address the identified factors were made by healthcare professionals and people with lived experience of chronic pain who had taken part in the mindfulness programme, also divided into physical, psychological and/or social domains. These included recommendations in the way patients are signposted to the programme, the content of the preprogramme letter and screening interview, inclusion of a taster session, provision of adequate equipment, teacher skill and experience, group size, programme content and support during the programme and at completion.

### 4.2 Significance of findings

Our findings support the theories by Holdsworth et al.^44^ and Banerjee et al.,^46^ applying the themes of physical engagement (session attendance and home practice), psychological engagement (including motivation, belief, commitment and intent) and social engagement (including therapeutic relationship with facilitator and group), to a population with chronic pain. Motivation to participate was affected by their understanding of the relationship between mindfulness and pain. This novel finding, reported in greater detail in our earlier paper,^47^ was crucial for engagement with the programme. Beliefs about the programme such as sceptical preconceptions and the expectation of pain‐relief were found, in the current study to be key barriers to engagement and have been described previously.^42^, ^54^, ^55^ The commitment of participants to bringing mindfulness into their life was demonstrated by incorporating mindfulness into their daily routine, enabling practices to become a habit, facilitating ongoing practice, also described in the literature.^56^ Participants who developed autonomy and empowerment through the programme expressed an intention to do activities benefiting them, such as maintaining a mindfulness practice. The sense of community they gained from the group and the support they gained from the programme facilitators contributed to the therapeutic relationship, affecting their engagement, also previously described.^41^, ^42^, ^56^

In addition to those defined by Holdsworth and Banerjee, we found a number of additional factors specific to a chronic pain population. There was a perception among some participants that the content and structure of the programme is based on a misunderstanding of chronic pain. They felt that the belief that pain is ‘all in the mind’ may be inherent within the programme itself. This experience of pain being disbelieved was a barrier to engagement, as reported previously.^42^

Increased self‐awareness was described in the current study as challenging for some participants, affecting engagement, and also found previously.^56^, ^57^ It may be a particular challenge of mindfulness because, as described by our participants, it involves bringing attention to uncomfortable experiences (including experiences of pain and uncomfortable psychological experiences). This is unlike other psychological approaches, such as CBT, that employ strategies designed to relieve discomfort.^58^ Increased self‐awareness may, in some situations be overwhelming, such as in the early stages of chronic‐pain related grief.^59^ In the earlier stages of the course of a chronic pain condition, practising mindfulness may increase the awareness of physical and emotional discomfort, resulting in a participant dropping out of the programme as an adaptive response. For some participants of our study, the increased self‐awareness gained through the programme enabled them to gain control over their thoughts, emotions and response to pain, facilitating ongoing practice, a finding also in the literature.^54^ The perception of control has been described as the extent to which participants feel empowered to take control of their own behaviours and responses,^54^ and as an increased sense of autonomy and competence in managing their pain.^56^ It may be that sense of autonomy and competence are motivating factors to ongoing practice, a suggestion supported by motivational theories such as self‐determination theory.^60^

Participants in our study with lived experience of chronic pain described mindfulness as being appropriate at a particular stage of their pain journey and that physical aspects may need to be met (e.g., optimising physical therapy) before being receptive to mindfulness. This is supported by Maslow's hierarchy of needs which suggests that a person is likely to seek to meet physiological needs before being motivated to take part in activities meeting psychological or spiritual needs.^61^ The transtheoretical model^62^ has been used to hypothesise that there are different stages of readiness of people with chronic pain to engage with self‐management, including at the precontemplation stage believing their condition is physical, requiring medical treatments and at the contemplation stage, being more likely to take part in psychological therapies as they contemplate self‐management as an alternative to medical interventions.^63^

Recommendations for modifying the programme to address the factors described above were divided into psychological, social and/or physical domains reflecting the multifactorial nature of chronic pain. Psychological modifications included having a mindfulness taster session before the screening interview to give information and experiences of short mindfulness practices. This, as well as carefully explaining the objective of the programme in the pre‐programme letter, and screening interview would help to address preconceptions and encourage more realistic expectations. The taster session has been suggested in some of the recent models^16^ but not others, and our study suggests that bringing it into standard practice may be of benefit.

We recommend considering the timing of participation in a mindfulness programme along the course of treatment for a chronic pain condition, determining whether the outcomes sought would be better met through, for example, a pain management programme, physiotherapy or occupational therapy. The stages of readiness can be identified using a questionnaire such as the Pain Stages of Change Questionnaire.^64^ The measure may predict uptake and engagement with self‐management approaches such as mindfulness and enable people with chronic pain to be matched to appropriate treatments depending on their degree of readiness.^65^, ^66^ It has also been found to predict outcomes with completers and noncompleters of pain self‐management programmes differing significantly on precontemplation, and contemplation scales.^63^ It is also acknowledged that the person's readiness to engage with a mindfulness programme may be difficult to judge, either by the person themselves or by the healthcare professional and that it would be acceptable to try an approach empirically recognising that it might not work, and not regarding this as a failure. If these approaches were available by referral from primary care, the conversation between the healthcare professional and patient about what might be the right treatment approach for them could be carried out by their GP; this was a social modification suggested by the healthcare professional participants in our study. This recommendation and support by the patient's GP could have an important impact on the patient with shared decision making promoting patient autonomy.^67^

Other social modifications included limiting the mindfulness group size to a maximum of 12–15 to enable a stronger sense of group cohesion. This supports the suggested group size for MBCT‐CP of 6–12 participants.^17^ Further social modifications not previously recommended include being able to opt‐in to reminder texts to be mindful and offering a support line or email address to ask questions or share difficulties between sessions. Connection to local community and online resources could provide additional support. Physical modifications included starting with practices of shorter duration and gradually building up to longer (30 min) practices, also recommended by Day^17^ as a way of approaching home practice.

Psycho–social modifications that would support the therapeutic relationship included particular skills and experience of the mindfulness programme teacher. The importance of the facilitator having their own personal practice of mindfulness is widely accepted good practice.^68^ Chronic pain self‐management research supports the role of empathy, support and quality of the provider–patient relationship in patient understanding, trust, motivation and treatment adherence.^69^

Socio‐physical modifications included acknowledging the experience and significance of having chronic pain as well has holding an inquiry session each week asking about difficulties encountered with home practice, held in a nonjudgemental way by a skilled teacher, already an aspect of some mindfulness programmes.^17^ Acknowledging the significance of having chronic pain can be further reinforced and addressed within the programme content by tailoring the practices and information to pain; a psycho‐physical modification. This has been addressed in some newly proposed tailored mindfulness programmes for chronic pain such as MBCT‐CP which includes psychoeducation on pain theory such as the ‘Gate Control Theory’, adapted from CBT for pain, from session 1.^17^ The Mindfulness for Health programme^16^ teaches participants to distinguish between primary pain (pain arising from injury or illness) and secondary pain (the mind's reaction to primary pain), thereby acknowledging that their pain is real and explaining how mindfulness can help; and includes teaching on pacing of activity, a technique commonly taught in CBT for pain. The MBCT‐CP and Mindfulness for Health programmes include much simpler movement exercises than the hatha yoga practices included in the original MBSR programme.^16^, ^17^ Both healthcare professionals and service users in our study suggested having mindfulness practices (during the session and for home practice) that are specifically for pain with options for different levels of ability and lengths of time, to increase autonomy and choice as participants are able to select the level that feels appropriate for them that day. This has not, to our knowledge, yet been suggested in the literature on tailoring mindfulness programmes for people with chronic pain. Other psycho‐physical modifications to further promote the sense of empowerment included encouraging participants to develop their own personal resources for ongoing practice (such as music that they listen to mindfully, or planned time in nature). Allocating time during the programme to build their own repertoire of mindfulness resources and the use of local as well as online mindfulness resources may provide support to continue practicing mindfulness after programme completion.

### 4.3 Strengths and limitations

Strengths included inclusion of people with lived experience of chronic pain and mindfulness (service users) in describing factors affecting engagement with, and generating suggestions for tailoring the programme. Using both individual and group processes enabled further suggestions to be generated though group discussion, with triangulation as service user ideas were taken to healthcare professionals and then their ideas taken back to service users.

Limitations included the low percentage of service users involved in the final stage of idea generation (17% of those invited). Previous studies on mindfulness for people with chronic pain demonstrate a wide variation in uptake ranging from 4% to 55%.^29^, ^39^ The small number of participants taking part may not be representative of the wider population of people with chronic pain and findings may not be generalisable. However, the findings are hypothesis‐generating and the ideas would need to be tested. The time period between delivery of the programme and data collection in 2013–2014 and the focus group meeting in 2017 may have contributed to the low attendance at the focus group meeting. Additionally, the delay between carrying out the study and publication of this paper is a limitation. Awareness and availability of mindfulness may have increased over the last 9 years which may impact on expectations and experience of the programme. However, research on mindfulness for people with chronic pain remains inconclusive and further research is recommended in the current NICE guidelines.^10^ Adapted mindfulness approaches for chronic pain are in the early stages and high drop‐out rates remain an issue.^22^ We therefore consider that our work remains topical and relevant today.

Additionally, the lead researcher (F. L. M. B.) being a mindfulness practitioner may have resulted in unknown biases. Members of the University of Aberdeen Division of Applied Health Sciences service user group who are people with chronic pain were consulted in the early stages of the research and contributed to the design of the study over two meetings but there was no patient and public involvement in analysis of data or authorship. Including the expertise of people with lived experience of chronic pain as a core part of the research team rather than just at the beginning was not possible due to no longer having contact with anyone from the University service user group.

### 4.4 Implications for research

The applicability of suggested modifications to the programme needs to be explored in practice. A phase two RCT, testing feasibility of recruitment, retention in the intervention and ability to collect patient outcomes beyond the end of the intervention, involving the modified programme is needed to assess whether there are improvements to uptake, engagement and retention.

It would also be valuable to identify which factors are relevant to engagement with the programme in general and which are specific to a chronic pain population. A similar study could be undertaken with a general population to compare findings. It could be hypothesised, from the definition of engagement proposed by Banerjee et al.,^46^ that beliefs and expectations about the mindfulness programme, quality of the teacher and group support, intention and commitment to make time to practice as well as external support enabling them to attend sessions, are not specific to chronic pain populations, whereas factors such as understanding the relationship between mindfulness and pain, disability limiting participation and not being believed for being in pain or false assumptions about the pain experience, would be specific to a chronic pain population. General factors could then be applied more widely to mindfulness programmes to optimise engagement.

Although the Pain Stages of Change Questionnaire^64^ has been found to be a useful measure to predict uptake and motivation to engage with self‐management approaches for pain, further research is needed to determine whether there are differences in the optimal stage to attend a mindfulness programme compared to CBT or other self‐management approaches for pain.

## 5 CONCLUSION

Physical, psychological and social factors affecting engagement with the mindfulness programme were identified and suggestions made to address these by service users and healthcare professionals. The stage of their pain journey and readiness to take part in a mindfulness programme versus other treatments for chronic pain should be considered by the patient and a healthcare practitioner such as their GP. Taster sessions and preprogramme information explaining the objective of the programme may foster more realistic expectations. Tailoring the programme to pain is important in acknowledging that the pain is real and options within activities may support patient choice and empowerment. Teacher skills and experience, as well as support during the programme are important. Some modifications have already been incorporated into adapted models of mindfulness for people with chronic pain, whereas some are new, though none have yet become standard practice. Further work needs to be done to take these forward into a trial, testing whether they do indeed increase accessibility and acceptability for people with chronic pain.

## CONFLICT OF INTEREST STATEMENT

The authors declare no conflict of interest.

## ETHICS STATEMENT

Ethical approval was received from the National Research Ethics Service North of Scotland (REC reference number 13/NS/0011) on 21 February 2013, NHS Highland Research, Development and Innovation on 1 March 2013 and the University of Aberdeen College Ethical Review Board on 12 April 2017.

## Supporting information

Supplementary information.

## Data Availability

The data that support the findings of this study are available from the corresponding author upon reasonable request.
