# Supplementary material for: Mindfulness for people with chronic pain: Factors affecting engagement and suggestions for programme optimisation
Source: Health Expect. 2023 Mar 12;26(3):1287–307. doi: 10.1111/hex.13745 (PMC10154871; doi:10.1111/hex.13745)
Supplement: Supplementary file 1 — Supplementary information. [file HEX-26--s001.pdf]

Appendix 1: Mindfulness programme content

The Mindfulness Programme

Attitudinal foundation of mindfulness practice

1. Non-judging
2. Patience
3. Beginner's mind
4. Trust
5. Non-striving
6. Acceptance
7. Letting go

Session plan

Week 1 Beginner's Mind

Exercises: **Walking meditation**, **raisin exercise**, **body scan**

Teaching: What mindfulness is, automatic pilot, mindful eating

Homework: Body scan daily, informal mindfulness practice, mindful eating

Week 2: Overcoming obstacles & non-striving

Exercises: **Mindfulness of the breath** (sitting meditation), **mindful movement**

Teaching: Mindfulness of the breath, non-striving

Homework: Body scan daily, mindfulness of the breath, informal mindfulness practice, pleasant events diary

Week 3: Staying present

Exercises: **Mindfulness of breath & then sounds**, **three-minute breathing space,** mindful movement, walking meditation

Teaching: Mindfulness in everyday life, walking meditation, patience

Homework: Body scan (alternate days), mindful movement, daily sitting meditation, 3-minute breathing space, unpleasant events diary

Week 4: Staying with what is difficult

Exercises: Meditation on a difficulty

Teaching: Working with difficulties, **3-minute breathing space in stressful situations**

Homework: Alternate days body scan & mindful movement or walking meditation, daily sitting meditation, bring awareness to difficulties and apply 3-minute breathing space

Week 5: Working with thoughts & emotions.

Exercises: **Sitting meditation: mindfulness of breath, thoughts & emotions.**

Teaching: Relating to thoughts, mindfulness of thoughts

Homework: Body scan, mindful movement & sitting meditation in different combinations, 3-minute breathing space integrated into daily life, bring awareness to thoughts & emotions in formal & informal practice, neutral events diary

Week 6: Silent practice

Exercises: Various practices above, **loving kindness practice**

Homework: Combination of practices learned, informal practice, stressful communication diary

Week 7: Taking care of ourselves

Exercises: Sitting meditation with **choiceless awareness**

Teaching: Taking care of ourselves, mindful communication

Homework: Combination of practices learned, choiceless awareness

Week 8: Preparing for the rest of life

Exercises: Combination of practices learned

Teaching: Preparing for life beyond the course & letting go

Homework: Nurture the seed of mindfulness in your life and allow it to grow

Appendix 2 Interview topic guide

Interview Schedule

1. Confirm consent to interview

2. Confirm consent to audio recording and transcribing

3. Assure of anonymisation of any quotes in any output

4. Confirm identity of participant

5. Turn on recorder:

‘I would like to hear about your experiences of the Mindfulness programme, including what it was like for you, what was difficult for you, and what you didn’t like about it. Is that okay for you?’

(The following themes can be covered in any order. These are prompts to be used to enable coverage of these themes (as appropriate) during the interview. The participant may bring their own themes that will also be discussed.)

**Questions**	**Probing questions (if needed)**	**Detailed options**<br><br>**(background information for interviewer)**
How was the Mindfulness programme for you?	Can you tell me more about your experience?	
	**What difficulties did you encounter in:**	

Did any difficulties come up for you during the Mindfulness programme? if so please tell me about them	Choosing to take part?	Logistical/ scheduling problems/ Fears/ Previous experiences/ influence of others/ beliefs & values
	Attending group sessions?	Group dynamic/ instructors/ logistical factors/ ability to do the practices/ effect of illness or pain/ motivation
	Doing home practice?	Time/ motivation/ support/ influence of others/ ability to do the practices/ effect of illness or pain
	Completing the course?	Time/ scheduling problems/ support/ influence of others/ experience of the programme/ effect of illness or pain/ motivation
	Continuing to practice after the course was over?	Time/ scheduling problems/ support/ influence of others/ experience of the programme/ effect of illness or pain/ motivation/ ability to do the practices
What feelings/ emotions came up for you during the programme?	How did you deal with these emotions?	
	How did the emotions affect your experience of the programme?	
	Did you feel supported?	By the instructor/ group/ your own family, friends or others?
What were your expectations of the Mindfulness programme at the beginning?<br><br>(see Q 41 in pre-programme questionnaire)	What outcomes did you expect?	Did these occur?
	What goals did you hope to achieve through the programme?	Were these achieved?

	Did you encounter any unexpected outcomes?	What were these?
What views do you have about health and the treatment of illness? (find out the participant’s values and mind/body view)	Such as use of medications, alternative & complementary medicine and self-help methods?	
	What is your view regarding meditation?	
	Why did you decide to take part in this study involving Mindfulness meditation? (refer to Q40 in pre-programme questionnaire)	
Do you have a spiritual practice/ view and how did that affect your experience?	How did this affect your experience?	
Do you intend to continue any of the practices learned during the programme?	Do you feel able to do the practices	Why do you feel unable/ able to do certain practices?
	Do you feel motivated to do the practices	What motivates you to do the practices/ why do you not feel motivated to do the practices?
	Do you expect to benefit from doing the practices	What benefits do you expect?
	Which practices will you continue?	When will you do these? Why will you continue these? Why will you not continue these?
How do you think the Mindfulness programme could be changed to be more acceptable/ practical for you?		
Is there anything else that you would like to share?		

This 'interview schedule' is merely the basis for a conversation: it is not intended to be prescriptive and certainly not limiting in the sense of overriding the expressed interests of the participant. It is important that the participant take the lead during the conversation. Often the resulting interview data are very different from what the researcher might have anticipated. (Biggerstaff and Thompson, 2008)

Appendix 3: Factors affecting engagement by stage of programme

Factors affecting engagement: physical

Enrolment	Attendance	Home practice	Continued practice	Summary
Difficulty sitting, standing or walking<br><br>Wanting better mobility	Unable to take part in some aspects of programme due to disability	Unable to do course reading due to being partially sighted		Disability
Wanting to control or reduce pain were reasons for enrolling. Dissatisfied with medication and wanting an alternative.	Inadequate management of pain or disability prior to programme	Practices helpful for pain management – using mindfulness practices at home to treat pain.	Inadequate management of pain or disability prior to programme	Inadequate management of pain or disability prior to programme
	Being able to adapt practices to their needs or level of ability was a facilitator. Participants expressed that having teaching centred around pain & pain-specific discussion would help engagement.	Shortening home practices made it possible to incorporate them. Informal practices easily fit into life.	Choosing easier practices/ practicing when it suits their lifestyle were facilitators to ongoing practice.	Adaptations to practices
	Singing bowl caused physical discomfort. Longer practices were uncomfortable. Mindful walking felt inappropriate.	More aware of pain during body scan practice		Discomfort with some practices
	Dizziness during sitting meditation, mindful walking bringing on chest pain	Worsened tinnitus/ sense of panic during home practice.		Adverse events
	Uncomfortable chairs	Home practice CD poor quality		Inadequate equipment

Transport problem				Transport problems
	Scheduling conflict	Lack of time/ having other practices to do	Lack of time/ prioritising other things	Scheduling conflicts

Factors affecting engagement: Psychological

Enrolment	Attendance	Home practice	Continued practice	Summary
Previous experience or ideas about mindfulness. Belief in mind-body connection. The participant or their friends or family sceptical about mindfulness.	Doubting the programme would benefit them		Knowing the benefits/ Seeing benefits of practicing in others	Preconceptions
Wanting better mobility was a reason for enrolling. Wanting an alternative to medication to manage pain. Wanting to reduce or control pain. Wanting improved sleep.	Expected programme to help more with pain. Disappointed at the lack of pain-specific discussion			Expectations
	Commitment to complete programme helped continue to attend. Pleasure from attending group sessions & finding practices enjoyable were facilitators to attending. Emotional discomfort during some practices a	Having a plan to practice increased motivation but being hard on self when failed did not. Practices became easier with familiarity and habit, incorporated into daily life (which took time). Wandering mind during practices was a reason for	Self-sabotage (not finding time to practice when stressed) vs becoming aware of being stressed being a motivator to practice. Lack of motivation without regular class, difficult to practice alone. Having the discipline to continue regular practice. Laziness a barrier to	Motivation to practice

	barrier to attending e.g. raisin exercise.	discontinuing the practice. Finding practice enjoyable was a motivator to doing it at home. Three minute breathing space easy to practice.	doing longer practices. Practicing became easier when a habit, incorporated into life - mindfulness becoming 'normal. Writing reminders in diary to be mindful helped ongoing practice. Change of routine a barrier to ongoing practice.	
	As a child, wanted to do what others could do but unable to due to disability. Felt unsupported by health professionals			Prior life experiences
Depression, anxiety or other mental health difficulties and wanting improved mental health and better sleep as goals from the programme. Anger or frustration at current situation.		Three-minute breathing space meditation practice helpful in managing symptoms of depression or anxiety. Practices help to manage feelings of anger and frustration. Practices helping sleep. Sense of permission to stop and take time for self. Mindfulness practices giving a sense of safety. Visualisation was useful for calming emotions.	Mindfulness useful for controlling emotions/ in stressful situations/ when in pain. Practices helping sleep. More relaxed, calm & peaceful after practicing. Making positive lifestyle changes as a result of programme. Realising the need for self-care enabled some to create space or make time for practicing. Learning to take responsibility for self resulted in empowerment and choosing to continue to practice	Management of emotions & mental health
	Could not see the connection between mindfulness & pain vs did not understand the relationship between	Realising the connection between accepting their pain, reduced tension and less pain. Using mindfulness practices to treat pain.	Mindfulness practices used to treat pain	Understanding the relationship between mindfulness & pain

	mindfulness and pain. It took time to get over initial resistance and understand the objective of the programme.			
		Programme felt like it was permission to stop and take time for self. Shortening home practices made it possible to incorporate them	Learning to take responsibility for own choices resulted in empowerment and choosing to continue to practice. Less fear of the future and better planning for it. Writing reminders in diary to be mindful. Choosing easier practices/ to practice when it suits their lifestyle. Making lifestyle changes as a result of programme to enable more practice.	Autonomy & empowerment

Factors affecting engagement: Social

Enrolment	Attendance	Home practice	Continued practice	Summary
Loneliness and isolation. Wanting to be with others who have a similar experience or condition	Felt less alone with their pain during group sessions. Sense of trust, community and being understood by other group members vs not being understood (assumed to be depressed due to pain).			Loneliness & sense of community
External demands from others e.g. Responsibility for a dependent	Support from family made attendance possible. External demands e.g. visitors staying	Support from programme teachers when struggling with home practice. Support from family enabled	Having to cope with external demands a facilitator to practicing. Practicing with	Support from others/ external demands

family member		some to have space to do home practice. Demands from others or lack of support a barrier to home practice.	partner helped continue.	
	Large group size caused discomfort			Group size
	Disruptive group members in first two sessions. Sharing in front of others difficult. Sense of trust developed in the group. Sense of safety/ Lack of sense of safety in group e.g. not safe to close eyes in meditation. Helpful not knowing each other's pain conditions.			Group dynamic
	Compassion for others in group and for self. Feeling a sense of injustice on behalf of others: inadequate care for people with chronic pain			Compassion & social justice

Factors affecting engagement: psycho-physical

Enrolment	Attendance	Home practice	Continued practice	Summary
	Feeling the benefit of learning to listen to the body rather than pushing through pain & suffering afterwards. Learning to accept pain.	Doing body scan when aware of feeling tense helped to relax	Developing a flexible response to pain rather than avoiding activities for fear of pain.	Learning to listen to the body & developing a flexible approach to pain

Factors affecting engagement: psychosocial

Enrolment	Attendance	Home practice	Continued practice	Summary
	Easier to practice in a group		Difficulty doing the practices alone. Refresher session or continuing to meet as a group after completion of programme would help support ongoing practice through practicing in a group, sharing responses to practices ad revisiting programme material	Group increased motivation to practice
	Teacher's ability to deal with the group was a facilitator to attendance	Support from programme teacher when struggling with home practice.		Teacher skill

Factors affecting engagement: socio-physical

Enrolment	Attendance	Home practice	Continued practice	Summary
	Feeling self-conscious and uncomfortable during group sessions at being unable to do some activities and opting out. Felt excluded from taking part in some activities in group sessions due to disability			Exclusion & fear of judgement due to disability
	Programme reinforced feeling of not being believed for being in pain		Being disbelieved by someone who recommended mindfulness (GP) was a barrier to	Pain not being believed

			ongoing practice	

Factors affecting engagement: psycho-socio-physical

Enrolment	Attendance	Home practice	Continued practice	Summary
Wanting better mobility was a reason for enrolling. Wanting to better understand pain was a reason for enrolling. Depression, anxiety or other mental health difficulties and wanting improved mental health was a goal from the programme. Belief in mind-body connection, relationship of stress to physical symptoms/ not keen to take medication. Wanting to control or alleviate pain. Dissatisfied with medication and wanting an alternative.	Physical treatment should be optimised before taking part in a mindfulness programme. Realising the connection between accepting their pain, reduced tension and less pain.			Stage of pain journey
	Felt moved on a spiritual level by practices. Feeling of safety during group sessions/ lack of feeling of safety during group sessions.	Programme gave permission to stop and take time for oneself. Greater awareness and appreciation of surroundings brought a quality of experiencing that was rewarding and enjoyable. Mindfulness practices gave a sense of safety	Felt more peaceful and sense of wellbeing from doing practices after programme completion. Greater awareness and appreciation of surroundings brought a quality of experiencing that was rewarding and enjoyable.	Awareness, appreciation & sanctuary

Appendix 4 Nominal group meeting information presented

Stakeholder meeting:

Designing a modified mindfulness programme

Aim

The aim is to explore, with stakeholders with expertise in the area of mindfulness and/ or chronic pain, potential modifications to the mindfulness programme to optimise its acceptability for chronic pain patients.

Research question

Can identified barriers to engagement be used to modify a mindfulness-based programme for chronic pain patients? (while ensuring the core components are retained)

With the objectives:

1. Increase uptake onto the programme
2. Increase engagement with the programme
3. Increase attendance at group sessions and minimise drop-out
4. Improve adherence to home practice
5. Increase continued mindfulness practice after programme completion

Stakeholder meeting methods

Study results will be presented to stakeholders describing barriers and facilitators to enrolling, engaging with and completing the programme (block 1). The modified nominal group technique will be used to enable stakeholders to give their individual suggestions for

modifications to the programme. This will be followed by small group discussions generating further suggested modifications which will be presented back to the larger group.

This will then be repeated for block 2 (barriers and facilitators to home practice and to continued practice after programme completion).

Aspects of the programme for potential modifications:

- Participant invitation (who, where)
- Information given to participants by letter
- Pre-programme interview with programme facilitator
- Mindfulness programme content – practices & theory taught
- Home practice resources

Stakeholders will then be asked, for each of the stages, to rank the suggested modifications, giving one to the most important, two to the second most and so on.

Following the meeting, the results of the ranking will be used to calculate average scores for each suggestion that has been ranked and a final list of the highest ranked suggestions will be generated and shared with stakeholders by email.

<u>Summary of Study Results</u>

Recruitment & attrition

Of 480 patients with chronic pain who were invited to take part, 42 consented to take part. 40 completed a pre-programme interview, 35 completed pre-programme questionnaires, 24 attended 1 or more mindfulness sessions. 10 dropped out of the programme (attending <4 sessions) and 14 completed the programme (attending 4 or more sessions).

Completers and non-completers were interviewed face-to-face immediately post-programme, and by telephone at 6 months post-programme. Pre-and post-programme questionnaires were completed collecting some qualitative as well as quantitative data.

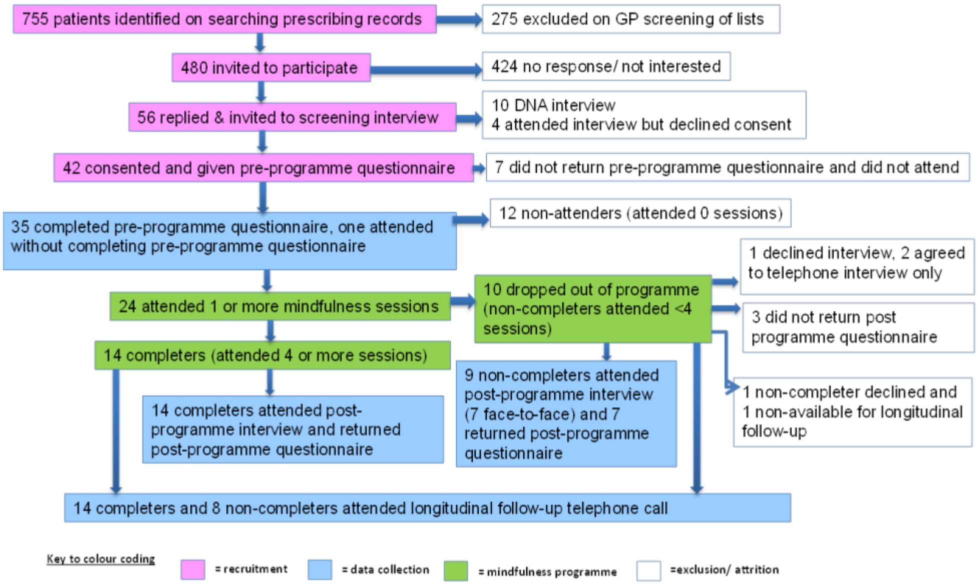

Qualitative data analysis

The following sources were analysed:

- Pre-programme interviews with programme facilitator (40 participants)
- Pre-programme questionnaires (35 participants) and post-programme questionnaires (21 participants)
- Post-programme interviews with lead researcher (21 participants)
- Six month follow-up telephone interviews with lead researcher (22 participants)

Emerging themes relevant to the research question are illustrated below. These will be presented at the stakeholder meeting.

Summary of high level themes

Qualitative analysis: High level themes

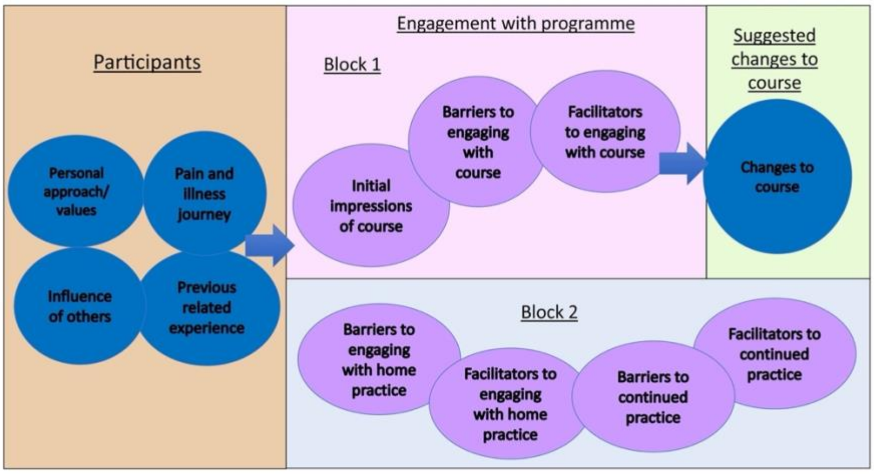

Participants

These themes relate to participant factors that may affect engagement with the mindfulness programme

Emerging themes: Participants

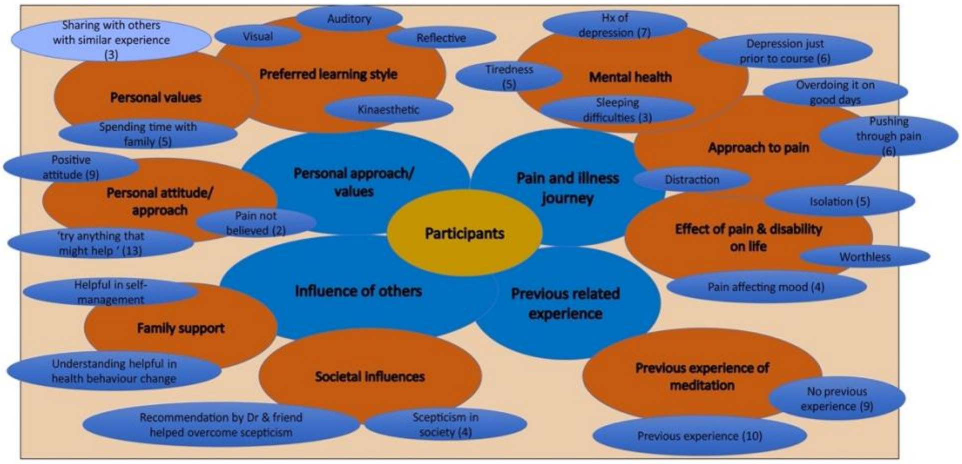

Enrolling on the programme

These themes relate to factors affecting enrolling, including concerns raised at the pre-programme interview.

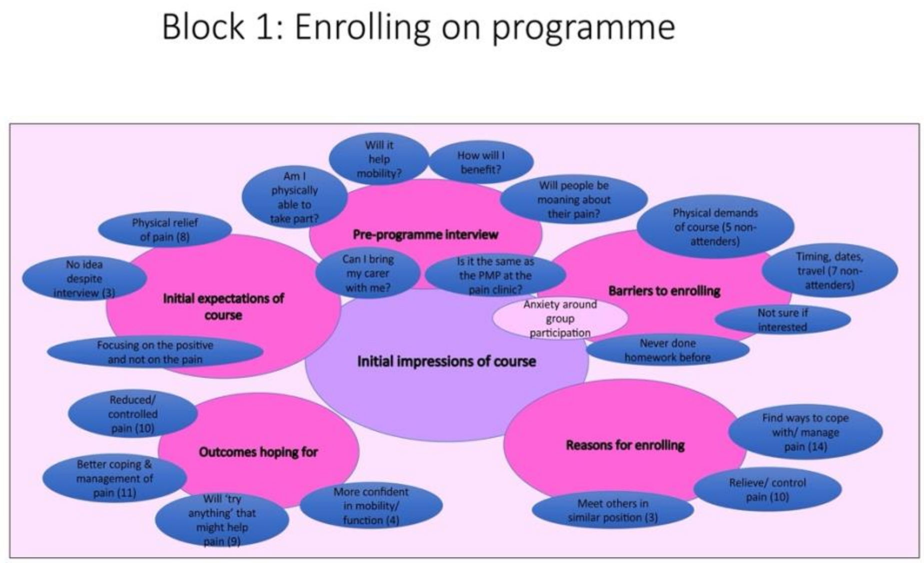

Barriers to engaging with and completing the programme

This includes barriers to attending sessions and engaging with the programme, as well as reasons for dropping out.

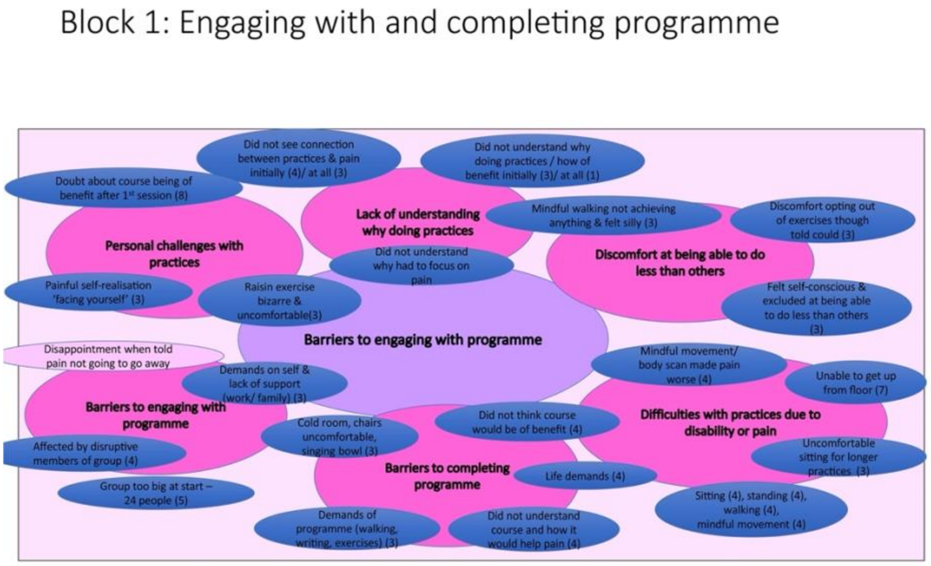

Facilitators to engaging with and completing the programme

This includes facilitators to attending sessions, engaging with and completing the programme

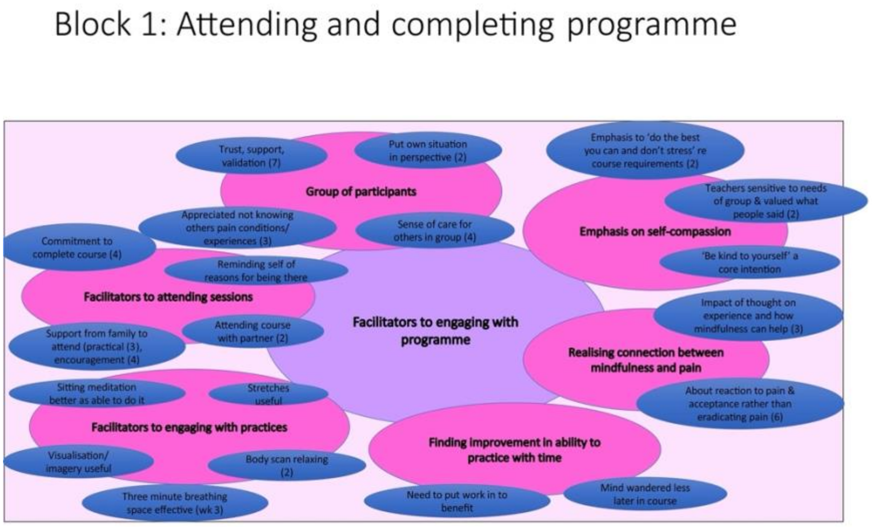

Suggested changes to the programme

Changes to the programme were suggested by participants. These are illustrated here.

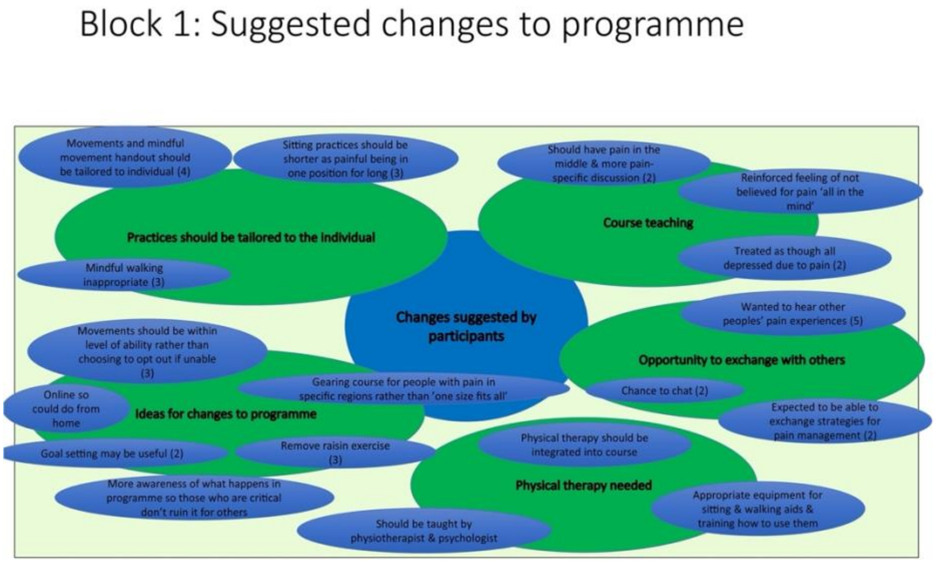

Barriers to home practice

These include the barriers to engaging with home practice

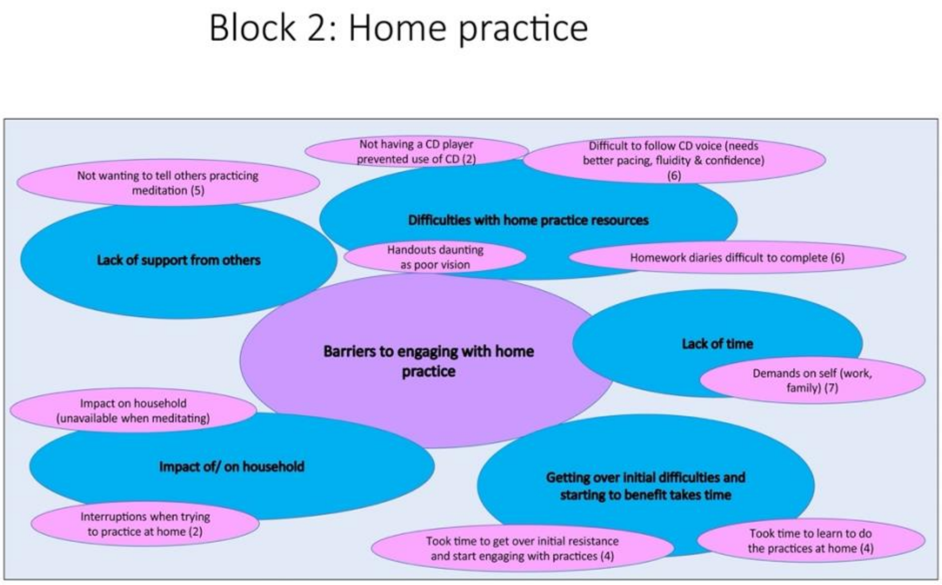

Facilitators to home practice

These include the facilitators to engaging with home practice

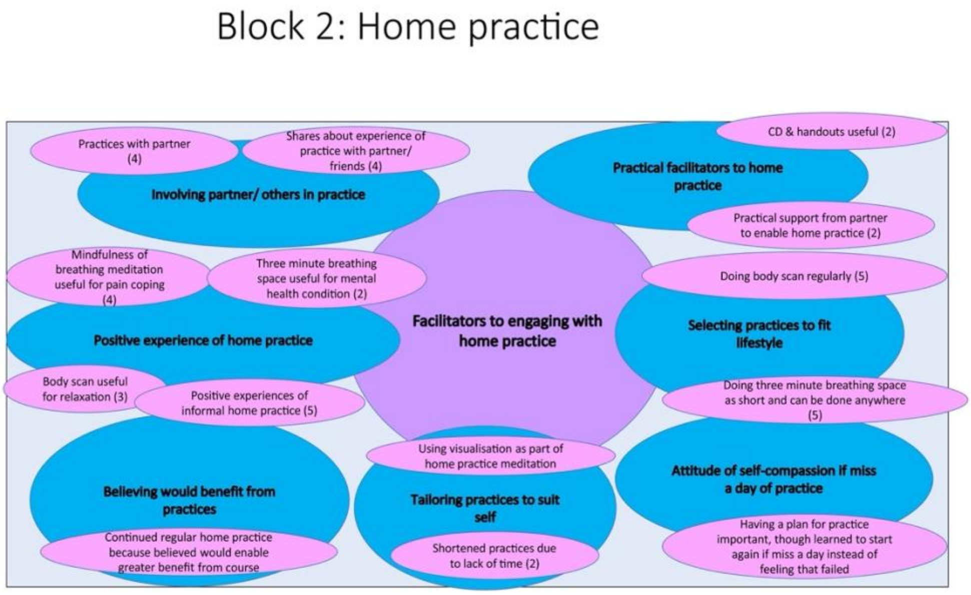

Barriers to continued practice

These include the barriers to continuing with mindfulness practice after course completion

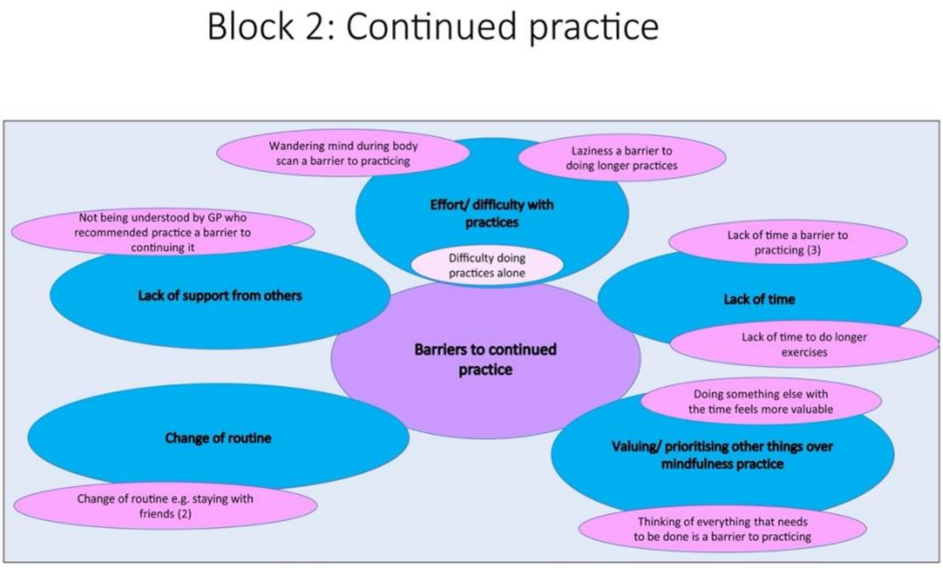

Facilitators to continued practice

These include the facilitators to continuing mindfulness practice after course completion

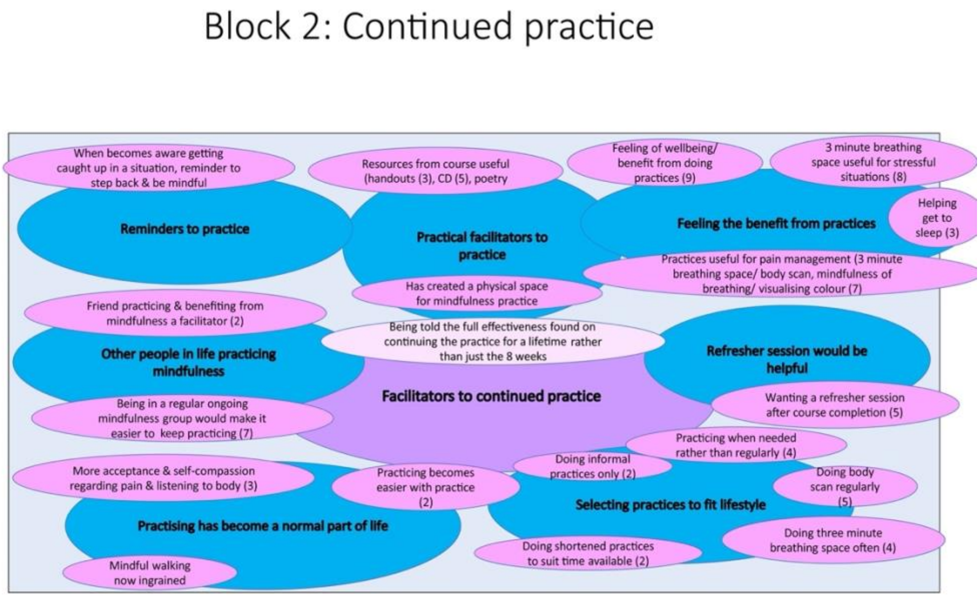

Description of aspects of the programme for potential modifications

- Participant invitation (who, where)
- Information given to participants by letter
- Pre-programme interview with programme facilitator
- Mindfulness programme content – practices & theory taught
- Home practice resources

1. Participant invitation (who, where)

Participant recruitment

Patients aged over 18 on repeated courses of analgesics and / or NSAIDs over last 3 months

Inclusion criteria:

- Adults aged 18 years or over
- Chronic pain of duration 3 months or longer; receiving regular prescribed medication for pain;
- Able to read and speak English fluently;
- Registered with one of the two eligible Fort William practices.

Exclusion criteria:

- Having a concurrent major psychiatric illness; diagnosis of personality disorder; acutely at risk of physical harm to self or to others; current known alcohol or drug addiction
- Pain caused by malignancy; red flags of serious underlying illness such as recent unexplained weight loss, fever, or sudden worsening of back pain; medical instability from heart or lung disease or other severe chronic medical conditions judged by the clinician to place the patient at risk of possible severe consequences of his/her disease
- Multiple recent falls or inability to stand independently
- Unable to give informed consent
- Illiterate, as they would not be able to complete the meditation diary
- Serious hearing or vision impairment that would preclude responding to questionnaires or participating in the meditation program
- Cognitive impairment

2. Information given to participants by letter

1. Invitation letter from GP surgery
   - Mindfulness is a new approach to managing pain and may help people find new ways of dealing with their pain.

2. Study information sheet
   - Mindfulness may help people find new ways of responding to their pain.
   - It is a treatment which addresses both physical health and emotional wellbeing
   - It involves learning to pay attention to what is going on in your body, your mind and the world around you. It is about living in the present, rather than dwelling in the past or anticipating the future

   What does the Mindfulness-based programme involve?
   - The programme will involve learning techniques such as focusing on the body, gentle movements and meditation while sitting on a chair. These will be taught in a group setting, and you will be encouraged to practice the techniques regularly at home.
   - There will be up to 30 participants taking part in the Mindfulness-based programme group sessions. The sessions will take place on one evening per week, and each session will be two hours long. There will also be an all-day session at the end of the programme.

3. Invitation letter to programme
   - The mindfulness-based programme will involve learning techniques to develop present moment awareness. These include relaxation and focusing on the body, gentle movements and meditation while sitting on a chair.

4. Mindfulness class information letter
   - Programme dates, Monday 5.30pm-7.30pm, location
   - Please bring with you a bottle of water, a mat (yoga mat or similar) if you have one and one or more cushions. Feel free to bring whatever you need for your own comfort during the class.
   - There will be chairs and you will not have to lie down or do movements if you do not feel comfortable to do them.
   - If you are unable to make it to a class, we request that you let the course facilitator know in advance if possible. She may then be able to get that week’s course materials to you. (number to phone to inform facilitator in advance)

3. Pre-programme interview with programme facilitator

Interview with programme facilitator: 10-30 mins, information on programme given, chance to ask questions and sign consent form

Main points covered during interview (#/40: number of interviews out of total 40 that covered this point):

1. What mindfulness is: Present moment awareness rather than worrying about the past or future. About being aware and awake to what is going on 23/40
    a. About being more kind & compassionate to ourselves 2/40
    b. Needs some work, may not get it at first but will after a while practicing 1/40

2. What course involves: Exercises involving awareness of your thoughts & feelings & body sensations. 13/40
    a. Attend to our breathing 11/40
    b. Build up to sitting for 30 minutes 2/40

3. Evidence: Mindfulness has been shown to help with anxiety, depression and chronic pain. 33/40
    a. Won't cure pain but will help people deal differently with pain 1/40
    b. Brains change and become happier 1/40

4. Benefits: Can help relaxation and help get to sleep 14/40
    a. Can help relax 6/40 (+14 above)

5. Practices taught: Mindful movement, mindful walking, sitting meditation, body scan 37/40

6. Movement: Involves gentle movements based on Tai Chi/ Chi Gong/yoga, not vigorous. It is about learning how far we can go and being aware of what our bodies are doing. 24/40
    a. Can sit and do them or do them in your imagination 13/40
    b. Can sit and do them 10/40
    c. Can sit it out 2/40

7. Walking: Involves gentle walking around the room. Whatever you can do, as best you can. To be aware of moving. 14/40
    a. Can sit and imagine walking 8/40
    b. Can sit it out 5/40

8. Body scan: Involves exploring what is going on in body. 8/40
    a. Can be done lying or sitting 20/40
    b. Done lying on floor 4/40
    c. Done sitting 2/40

9. Pain management: We will look at pacing 8/40
    a. You will learn to relate differently to pain 5/40
    b. You will learn a more flexible approach to pain (breathe into it and know when reached an edge and need to take a painkiller) 4/40
    c. You will gain more acceptance of pain 2/40
    d. It will help pain management 4/40

10. Group: It is not a psychotherapy group. The facilitators lead you through the exercises and then ask what you found during the exercises. You won't have to speak if you don't want to. We will do some work in pairs. The facilitators are available at the beginning & end for private questions 27/40
    a. We don't go into life issues or problems 21/40
    b. We don't ask about pain conditions 2/40

11. Homework: You will be given a CD & handouts. 27/40
    a. This involves practicing what we did in class 14/40
    b. You will get benefit from doing home practice 7/40 (1/40 – practicing helps, it's not going to happen in the first couple of weeks, it's a case of keep practicing).
    c. There are forms/ diaries to complete 3/40
    d. We ask you to practice 6/7 days per week for 20 mins/ day 1/40

4. Mindfulness programme & home practice resources

Sent as separate attachment to email

- Mindfulness Scotland Mindfulness Approaches 8 week programme course handbook (given as handouts each week to participants)
- Mindful movement handout

Appendix 5 Suggested modifications list

Please rank up to 5 ideas for each of the 8 categories below. Consider the ideas that you think are most important and please only rank the ideas that you support.

I have attached the ranking sheet. Ranking sheets can be kept anonymous, sent to my postal address, or emailed to me. There is a space on the ranking sheet for comments.

Category 1: Enrolment

Publicity and promotion of Mindfulness courses

1. Convince GPs of the benefits of mindfulness courses so that they promote mindfulness to appropriate pain patients
2. Publicise mindfulness courses
3. Mindfulness on prescription
4. Educate wider healthcare professionals about mindfulness e.g. practice nurses
5. Use a model of pain when promoting the course that acknowledges peoples pain and addresses the mind/body dichotomy (to overcome the problem with people thinking mindfulness for pain means we think pain is all I their mind)

Where to enrol from

1. Personal approach to invitation & enrolling: participation in the programme to be recommended and supported by someone who has an ongoing professional relationship with the patient e.g. their GP or pain specialist
2. Enrol from pain service (e.g. pain cinic) rather than from general practice
3. Have a mindfulness taster session within pain service that could then feed into a mindfulness course if people engage with the taster session (to minimise drop-out from the mindfulness course)
4. Have an introduction to self-management course first (e.g. 3 x 2 hour sessions) that can then feed into an 8 week mindfulness course for people who are suitable.

Category 2: Pre-programme letter

Format of pre-programme information letter

1. Format of letter – use infographics (visual cues) to communicate the information
2. Have video links to clips that can be watched rather than/ or to compliment written info

Content of pre-programme information letter

1. The mindfulness course offers a new approach to managing pain by changing their relationship to their pain (rather than changing their pain)
2. The mindfulness course is about improving quality of life (don’t say it is for managing pain).
3. It involves group sessions of 10 to 15 people (rather than up to 30 participants)
4. It may take a few weeks to feel a benefit from the course but effort will pay off
5. Time is required for home practice as well as attending sessions
6. The course involves learning a lifelong technique and the longer it is practiced the greater the benefits
7. Testimonials with positive experiences of programme graduates

<u>Use of language in pre-programme letter & screening interview</u>

1. Translate concepts into everyday language. (See MBCT handout for an example of this)
2. De-medicalise language, don’t use language associated with pathology e.g. don’t say ‘mindfulness helps depression & anxiety’, use everyday language instead.
3. Avoid emphasising certain feelings as being good or bad e.g. rather than using the word depression, call it an ‘intense emotional experience’. Take care around how we relate/ respond to positive & negative experiences (labelling it as depression may be emphasising it being wrong or bad)
4. Avoid terms such as ‘mental health and physical pain’, or ‘physical health and emotional wellbeing’ as these emphasise the dichotomy separating mind & body.

Category 3: Screening interview

<u>Participant stratification & selection at screening interview</u>

1. Be clear about what the patient wants to achieve – if it is pain control then it would not be appropriate to enrol onto mindfulness programme. If it is to help managing activity etc then consider enrolling on pain management programme.
2. Consider the emotional functioning of potential participants when judging suitability for the course (within applied mental health, mindfulness (MBCT) is used for relapse prevention)
3. Have clear criteria to screen out those with unrealistic expectations
4. Stratify by functional level (so people of similar functional levels are in the programme together) & have a different course structure depending on functional level
5. Identify participants who may require additional support at the pre-programme stage & during the programme e.g. people from lower socioeconomic group/ those who find coming into a group setting challenging, and offer them more support (e.g. Support phone calls or 1 to 1 time)

Information given at screening interview

1. It is about changing their relationship to their pain rather than changing their pain
2. It may take a few weeks to feel a benefit from the course but effort will pay off
3. Time is required for home practice as well as attending sessions
4. Explain the benefit and also the difficulties of home practice
5. The course involves learning a lifelong technique and the longer it is practiced the greater the benefits
6. Describe the movement as ‘gentle movements’ rather than as Tai Chi/ Qi gong/ Yoga (people may find these off-putting or strange).
7. Demystify the use of the singing bowl – explain that there is nothing magical about it, it is just a practical way of marking the end of a practice.
8. Don’t ask people to bring things with them to the course (e.g. yoga mats & equipment) as this may put people off coming if it is difficult to arrange to bring these things (may be coming straight from work etc). Provide equipment instead.

Category 4: Engaging with the programme

Pre-course group session

1. Have a mindfulness taster session to prepare for taking part in the programme
2. Include sharing of pain stories and strategies between participants (‘pain talk’) in a pre-course group session.
3. Have a pain-education class prior to the 8 week course including pain self-management

Course content (non pain-specific)

1. Remove the raisin exercise
2. Have a ‘chocolate exercise’ instead of a raisin exercise
3. Keep longer practices as that is where the most learning and gain is – when uncomfortable & staying with the discomfort
4. Course handbook name and content emphasising that it is a practice for life not just an eight week course (e.g. ‘MBCT for Life’ is the name of an MBCT handbook)

Course practicalities

1. Have a smaller group e.g. max 12-15 this will reduce drop-out rates as it is more personal.

2. Invite people to attend the whole course with a family member or a friend
3. Have a tea break as a chance for people to chat and share experiences (e.g. pain experiences) if they want to
4. Equipment: have chairs rather than mats so it is not focused on yoga & lying down
5. Have one session of the course open for family/ friend of participant to attend

Teacher requirements

1. Have the same person conducting the screening interviews as delivering the course (and that person should be an experienced mindfulness teacher)
2. Important that the teachers of the course have their own personal practice of mindfulness
3. Teachers need to have had adequate training and especially be skilled at leading ‘the inquiry’ process and relating it to pain

Category 5: Tailoring the programme to pain

Tailor course to pain

1. Include pain-relevant content. MBCT has well-defined exercises around thoughts & feelings. MBSR doesn’t have this for pain. This could be developed and incorporated into the programme.
2. Have recordings of home practices specifically for pain e.g. Jon Kabat Zinn’s Mindfulness for Pain CDs
3. Have shorter practices only (up to 15 minutes) and not longer (30 minute) practices
4. Map exercises onto those with least ability (so everyone does what the least able people are able to do)
5. Have 2 levels demonstrated for each exercise (one that requires more physical ability and one that requires less) – so that both options are as valid and normal (rather than one being an ‘opting out’)
6. Build up duration of meditation practices gradually
7. Acknowledge person’s condition during programme exercises (specifically state that during practices it is fine to stand up/ move/ lie down whenever needed in order to best manage their own pain)
8. Normalise/ praise looking after oneself e.g. give praise to the person who is the first to sit down when doing the walking exercise or movement practices.

Category 6: Support

Support between sessions

1. Patient's GP to be made aware that their pain may increase during the programme (as we are asking people to get closer to their pain) so that they expect this and can provide support if required
2. Offer 'retrieval classes' for people who miss a session (e.g. one-to one classes with facilitator to enable the participant to catch up on what was covered in the session)
3. Support phone-call between sessions
4. Have a support line for people to call during the week regarding home practice difficulties
5. Email address for questions between sessions
6. Sending reminder texts to participants to be mindful between sessions
7. Have a group blog or online group/ e.g. Facebook group where the participants can share their experiences and connect during the week.
8. Send out information each week with information from flip charts that is personalised for the group (e.g. containing personalised group learning from the inquiry process that week)

User involvement

1. User involvement in teaching – invite people who have completed the 8 week course who have chronic pain to attend one session of a new course to share their experiences of the course with participants
2. Have previous graduates (participants who have chronic pain and have completed an 8 week course) to lead or co-facilitate the group

Category 7: Home practice

Supporting home practice during group sessions

1. Have explicit exploration of the obstacles to home practice during the course, and generate ideas as a group as to how to address the obstacles.
2. Have an inquiry session during the weekly group sessions specifically asking about the participants' experiences of the home practice done that week
3. Include didactic sessions on habit & routine forming (regarding home practice), dealing with flare-ups & dealing with change
4. Discuss the usefulness of having a routine for home practice & reminders to practice

Home practice practicalities

1. Frame the 30 minute practices as being about staying with what is difficult. State that it is fine to move during the practice if in pain but to try to keep to the whole length.
2. Put a time limit on the duration of home practices recommended e.g. 15 mins.
3. To not recommend practicing 6 out of 7 days if that is not necessary or realistic (suggesting practicing 6 out of 7 days may result in people feeling guilty or stopping practicing if they don't manage to keep to that).
4. Don't call home practice homework
5. Have a variety of modalities for home practice resources e.g. app, online, CDs, DVD of mindful movement exercises
6. Have different levels within home practice resources (e.g. level 1 & level 2) for different levels of physical ability/ different lengths of time of practices.
7. Familiar voice on home practice resources e.g. voice of the teacher of the course
8. Encourage people to do their home practice with family or friends

Category 8: Continued practice

Supporting Continued practice

1. Have an all-day session during the course that is open to people who have completed 8 week courses
2. Build a follow-up group into the programme so that people continue to meet and practice after completing the 8 week course e.g. in a community hall
3. Online resources or links to apps/ websites to support practice after completing the course (e.g. 'Insight timer' app)
4. Make use of local community resources after course completion e.g. ongoing mindfulness groups that participants can feed into after course completion

Appendix 6 Suggestions for Mindfulness Programme Ranking Sheet

Please rank up to 5 suggestions for each category based on their importance (first column) and in the 2nd column please consider how feasible that change would be. Rank only the ideas that you support.

Category 1: Enrolment

Ranking	Idea ranked on Importance	Feasibility of implementing

1		
2		
3		
4		
5		

Category 2: Pre-programme letter

Ranking	Idea ranked on Importance	Feasibility of implementing
1		
2		
3		
4		
5		

Category 3: Screening interview

Ranking	Idea ranked on Importance	Feasibility of implementing
1		
2		
3		
4		
5		

Category 4: Engaging with the programme

Ranking	Idea ranked on Importance	Feasibility of implementing
1		
2		
3		
4		
5		

Category 5: Tailoring the programme to pain

Ranking	Idea ranked on Importance	Feasibility of implementing
1		
2		
3		
4		
5		

Category 6: Support

Ranking	Idea ranked on Importance	Feasibility of implementing
1		
2		
3		
4		

5		

Category 7: Home practice

Ranking	Idea ranked on Importance	Feasibility of implementing
1		
2		
3		
4		
5		

Category 8: Continued practice

Ranking	Idea ranked on Importance	Feasibility of implementing

1		
2		
3		
4		
5		

Any other comments or suggestions

Appendix 7 Postal list of ideas

Pt ID

Ideas for Changes to the Mindfulness Programme

The ideas discussed at the meeting on 7th December are listed below. Please tick whether or not you think they will be useful.

Ideas from specialists that participants agreed with

This section includes ideas that were suggested by programme participants, pain specialists and mindfulness teachers that attendees at the meeting on 7th December agreed with.

Category 1: Changes to the pre-programme letter and interview

	**Pre-programme information (letter and interview)**	Useful	Not useful	Don't know
1	State that mindfulness course is about changing the relationship to pain and improving quality of life (rather than changing the pain)			
2	State that time is required for home practice but effort will pay off			
3	State that it may take a few weeks to benefit, it is a lifelong technique and benefits increase with practice			
4	Use everyday language, not medical or technical terms e.g. Describe the movement as 'gentle movement', not 'yoga'/ 'tai chi'/ 'qi gong'.			

	**Pre-programme letter**	Useful	Not useful	Don't know
1	Include video-links to clips of patient stories and experiences of the mindfulness course and mindfulness exercises			

2	Use pictures to describe information			
3	Include quotes from people with chronic pain who have completed a mindfulness course			

	**Pre-programme interview**	Useful	Not useful	Don't know
1	Establish where the patient is on their pain journey (whether they have had physical treatment), what their expectations are and what they want from the course. Redirect (e.g. to pain management programme or pain clinic) if mindfulness is not appropriate to meet their needs.			
2	Have the same person conducting the pre-programme interview as teaching the course			
3	Don't ask people to bring equipment to the course, provide it instead (mats, cushions etc.)			

Category 2: Changes to the mindfulness programme

	**Changes to programme**	Useful	Not useful	Don't know
1	Have a mindfulness taster session before the course (to explain the background, research and what mindfulness is). This could be delivered to a larger group before the pre-programme interview			
2	Have a smaller mindfulness course group size (e.g. Maximum 12-15 people)			
3	Have a short tea break during the group sessions as a chance to chat			

4	Could offer one session for participants to bring a family member or friend to learn about what they have been doing on the course (e.g. week 7)			
5	Don't have mindful walking at the start of the programme, have it later on			

	**Tailoring the programme to pain**	Useful	Not useful	Don't know
1	Content of the programme needs to be relevant to pain (including having home practice recordings specifically for pain)			
2	Acknowledge participants pain and emphasise doing what is needed to manage pain and look after self during practices			
3	Build up to the 30 minute practices gradually (keep the 30 minute practices in, state that this is about staying with what is difficult)			
4	Have 2 or 3 options demonstrated for each exercise for different levels of ability. Participants can choose whatever option is most acceptable to them that day.			

	**Support**	Useful	Not useful	Don't know
1	Send reminder texts to be mindful between sessions (e.g. 2x per week), participants to opt in if they want this			
2	Have a support line or email address for home practice difficulties			

3	Phone call from course teacher between sessions (participants can opt in if they want this)			
3	People with chronic pain who have completed a mindfulness course to attend one session of the new course to share their experiences			
4	People with chronic pain who have completed a mindfulness course to assist in facilitating the course			

Category 3: Changes to help home practice

	**Changes to help home practice**	Useful	Not useful	Don't know
1	Explore obstacles to home practice as a group and generate ideas to overcome the obstacles			
2	Discuss routine/ habit forming in doing home practice and having reminders to practice			
3	Have a variety of modalities of home practice resources e.g. CD, DVD, app, online, handouts			
4	Have different options within home practice resources for different levels of physical ability and for different lengths of time e.g. Options 1, 2 and 3 for each exercise			

Category 4: Changes to help continued practice after completion of course

	**Changes to help to continue to practice**	Useful	Not useful	Don't know
1	Build a follow-up group into the programme that continue to meet and practice together after course completion			
2	Online resources to support ongoing practice			
3	Make use of local community resources e.g. local mindfulness group			
4	Have an all-day session during course open to people who have completed mindfulness courses			

Ideas from specialists that participants did not agree with

This section includes ideas that were suggested by pain specialists and mindfulness teachers that participants at the meeting on 7th December did not think would be useful.

	**Changes that participants did not think would be useful**	Useful	Not useful	Don't know
1	Have people with similar levels of physical ability in same course e.g. one course for people who are less physically able and one for people who are more physically able			
2	Include sharing of peoples' pain stories and experiences in mindfulness taster session			

3	Have a pain self-management class/ pain education course prior to the mindfulness course			
4	Have short practices only (up to 15 minutes)			
5	Have a familiar voice on home practice resources (e.g. course facilitator)			
6	Have tea break during group sessions long enough to share pain stories and experiences			
7	Don't recommend daily home practice (or 6 days a week)			

<u>Ideas from participants</u>

This section includes new ideas generated by participants at the meeting on 7th December.

Category 1: Changes to the pre-programme interview

	**Pre-programme interview**	Useful	Not useful	Don't know
1	Ask for commitment (to attending 6 or 8 sessions) as takes time to see benefit from the programme			
2	Acknowledge scepticism and ask people to try to be open minded for the 8 weeks of the course			

Category 2: Changes to the mindfulness programme

	**Changes to programme**	Useful	Not useful	Don't know

1	Have some mindfulness courses for specific types of pain e.g. people with back pain, and some generalised courses for people with chronic pain.			
2	Move the raisin exercise to later on in the programme rather than week 1			
3	Raisin exercise: Use another sensory object instead of a raisin e.g. a pine cone			
4	Singing bowl could be changed to music or a bell if there is discomfort or stigma with using the singing bowl			
5	If unable to do a practice physically, being asked to do it in your imagination may be difficult or a negative experience. Instead of asking people to do it in their imagination, give them 2 or 3 options for different levels of ability and ask them to choose what they feel most comfortable with that day			
6	Qualities of the teacher of the course are important: being calm, confident, experienced, being able to deal with difficult emotions in the group, and for participants to be able to trust them are all important qualities			

Category 4: Changes to help continued practice after completion of course

	**Changes to help to continue to practice**	Useful	Not useful	Don't know
1	Have a refresher session after completing course			
2	Continue reminder texts to be mindful for 6 weeks after course completion (participants to opt-in to receive these)			
3	Encourage participants to develop their own resources to support their ongoing mindfulness practice (e.g. music/ nature)			

Please share any other comments or suggestions here

___

___

___

___

___

___

___
